# 7-Prenyloxycoumarins as Promising Antileishmanial Agents: In Vitro, In Vivo, and In Silico Evaluation Against *Leishmania amazonensis*

**DOI:** 10.3390/ph19030426

**Published:** 2026-03-05

**Authors:** Dirlei Nico, Daniel Clemente de Moraes, Anna Claudia Silva, Igor Nunes Taveira, Yasmin da Silva Fontes, Rosangela Sabbatini Capella Lopes, Cláudio Cerqueira Lopes, Antonio Ferreira-Pereira

**Affiliations:** 1 Departamento de Microbiologia Geral, Instituto de Microbiologia Paulo de Góes, Universidade Federal do Rio de Janeiro, Rio de Janeiro 21941-902, RJ, Brazil; dirlei@micro.ufrj.br (D.N.); danielcmoraes@micro.ufrj.br (D.C.d.M.);; 2 Laboratório de Síntese e Análise de Produtos Estratégicos, Instituto de Química, Universidade Federal do Rio de Janeiro, Rio de Janeiro 21941-909, RJ, Brazil; anna.silva@ufrj.br (A.C.S.);; 3 Departamento de Microbiologia, Imunologia e Parasitologia, Faculdade de Ciências Médicas, Universidade do Estado do Rio de Janeiro, Rio de Janeiro 20550-170, RJ, Brazil

**Keywords:** altissimacoumarin D, antileishmanial, coumarins, *Leishmania*, spermidine synthase

## Abstract

**Background/Objectives:** Leishmaniasis remains a major neglected tropical disease, and current chemotherapeutic options are limited by toxicity and resistance in *Leishmania* species, including *L. amazonensis*. Prenylated coumarins have emerged as promising bioactive scaffolds. Altissimacoumarin D and its analogues inhibit fungal efflux pumps associated with resistance. However, their antileishmanial potential and mechanisms of action remain unclear. Here, we evaluated the in vitro, in vivo, and in silico effects of altissimacoumarin D and seven analogues against *L. amazonensis*. **Methods:** In vitro assays were performed to identify active compounds and assess toxicity in keratinocytes. In vivo experiments in hamsters evaluated antileishmanial activity and renal and hepatic toxicity. In silico analyses were conducted to investigate the mechanism of action of the substances. **Results:** In vitro assays showed that ACS47, ACS48, and ACS51 were the most active and safe compounds. In a hamster infection model, daily administration of ACS47 and ACS48 (2.5 mg/kg) significantly reduced parasite burden and lesion size, while maintaining normal renal and hepatic biochemical parameters. Histological analysis correlated reduced lesion size with marked decreases in amastigote density. Based on in silico analysis, spermidine synthase was supported as a plausible molecular target. **Conclusions:** Collectively, these findings identify ACS47 and ACS48 as promising lead compounds for future antileishmanial drug development.

## 1. Introduction

Leishmaniasis is one of the most significant neglected tropical diseases, with approximately one million new cases reported annually worldwide [1]. In the Americas, *Leishmania amazonensis* is a major etiologic agent of cutaneous and diffuse disease, often associated with therapeutic failure [2]. Current treatment relies on pentavalent antimonials, amphotericin B, and miltefosine; however, their use is limited by high toxicity, long treatment regimens, and the emergence of resistant strains, highlighting the urgent need for safer and more effective therapies [3].

Natural products play a central role in drug discovery because of their remarkable structural diversity and biological versatility [4]. Among them, coumarins constitute an important family of phenolic lactones widely distributed in plant species [5]. These substances display diverse biological activities, including antibacterial [6], antifungal [7], and antiparasitic [8] properties, making them attractive scaffolds for the development of new pharmacological agents. However, despite increasing interest in coumarins as antiparasitic agents, mechanistic investigations remain limited, particularly regarding prenylated derivatives and their structure–activity relationships in *L. amazonensis*.

Structural modifications of the coumarin core can significantly influence their pharmacokinetic and pharmacodynamic properties, enhancing their activity and toxicity profile [9]. Prenylation, i.e., the addition of an isoprenoid chain such as a prenyl or geranyl group, is a key modification that increases lipophilicity, thereby improving membrane permeability and interaction with hydrophobic biological targets [10]. Accordingly, prenylated coumarins isolated from *Ferulago angulata* and *Prangos asperula* have shown promising activity against *L. major* [11]. Recent evidence indicates that coumarins may act on multiple targets in *Leishmania*, including H_2_O_2_ production, gp63, and pteridine metabolism [12,13,14]. Nonetheless, it is unclear how variations in side-chain length and substitution pattern in 7-prenyloxycoumarins influence antiparasitic activity and target engagement.

In a previous study from our group, prenylated coumarins analogous to altissimacoumarin D reversed azole resistance in *Candida albicans* and, in some cases, directly inhibited fungal growth [15]. Given that both *Candida* and *Leishmania* share eukaryotic cellular organization and contain ergosterol in the plasma membrane, we hypothesized that structural variations in 7-prenyloxycoumarins could modulate antileishmanial activity, possibly through interference with essential metabolic pathways in *L. amazonensis*. Therefore, this study aims to investigate the in vitro, in vivo, and in silico effects of altissimacoumarin D and its analogues against *L. amazonensis*, in order to correlate their structural features with antiparasitic activity, and to elucidate their mechanism of action, supporting the development of novel drugs for leishmaniasis therapy.

## 2. Results

### 2.1. Evaluation of Coumarins’ Effects on Promastigote Viability

**Figure 1:**
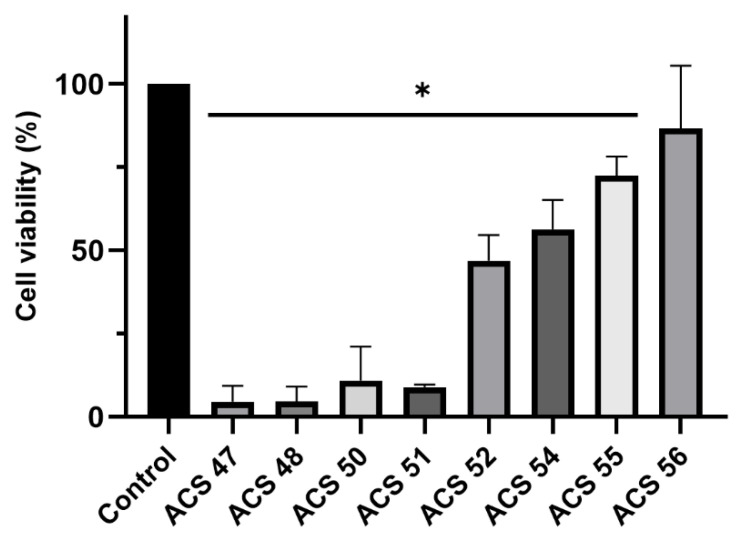
Evaluation of antileishmanial activity of prenyloxycoumarins against *L. amazonensis*. Cells were incubated with compounds (ACS47-56) at 100 μg/mL for 72 h. Cell viability was measured by MTT reduction assay. Data are expressed as mean ± standard deviation of three independent assays. (*) *p* < 0.05.

To evaluate whether compounds impair promastigote viability, compounds were screened at 100 μg/mL against *L. amazonensis*. As shown in Figure 1, all compounds except ACS56 exhibited antileishmanial activity. ACS47, ACS48, ACS50, ACS51, ACS52, ACS54, and ACS55 decreased *Leishmania* viability by 95.5%, 95.4%, 89.1%, 91.0%, 53.0%, 43.6%, and 27.5%, respectively.

**Figure 2:**
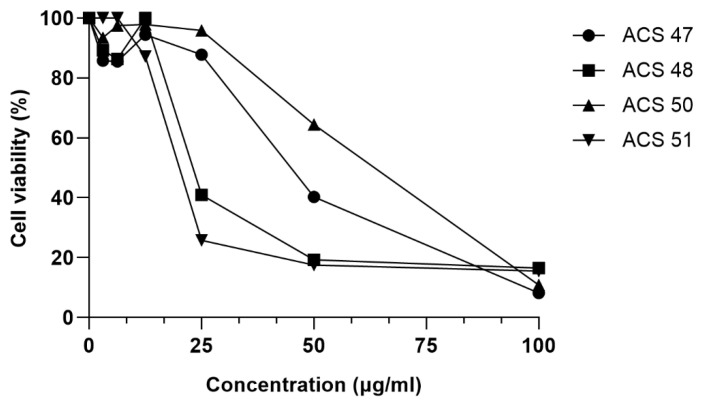
Evaluation of antileishmanial activity of ACS47, ACS48, ACS50, and ACS51 against *L. amazonensis*. Cells were incubated with compounds at serial concentrations (100–3.125 μg/mL) for 72 h. Cell viability was measured by MTT reduction assay.

ACS47, ACS48, ACS50, and ACS51 were further tested at serial concentrations. ACS47 showed antileishmanial activity at 50 μg/mL, reducing viability by 59.7%. At 25 μg/mL, ACS48 decreased viability by 59.0%. ACS50 was the least active compound, inhibiting *L. amazonensis* viability by 35.6% at 50 μg/mL, and was therefore excluded from further experiments. On the other hand, ACS51 was the most active coumarin, decreasing viability by 74.2% at 25 μg/mL. No activity was observed at lower concentrations (Figure 2).

### 2.2. In Vitro Toxicity Against HaCaT Cells

**Figure 3:**
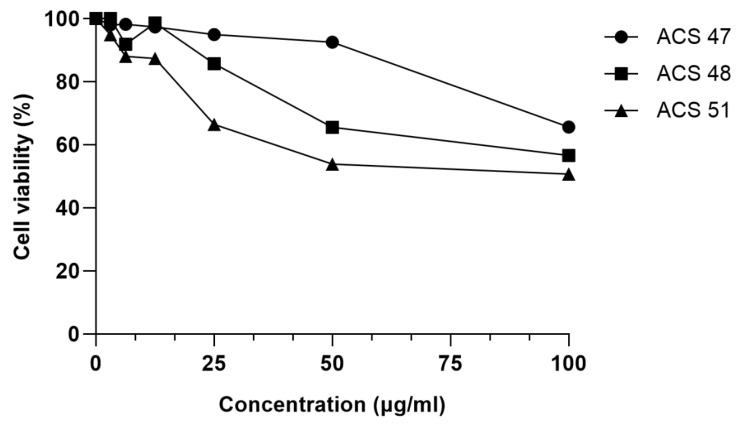
The in vitro toxicity of coumarins on human keratinocyte cells (HaCaT). Cells were incubated in the presence of coumarins at 100–3.125 μg/mL at 5% CO_2_ atmosphere for 48 h at 37 °C. Cell viability was then measured by MTT reduction assay.

The toxicity of ACS47, ACS8, and ACS51 at serial concentrations was evaluated in HaCaT cells. ACS47 showed toxicity only at 100 μg/mL, decreasing cell viability by 34.4%. ACS48 reduced HaCaT viability by 14.3% at 25 μg/mL, 34.4% at 50 μg/mL, and 43.4% at 100 μg/mL. ACS51 decreased cell viability by 33.6–49.3% at 25–100 μg/mL (Figure 3).

### 2.3. Assessment of Evolution of Lesions and Parasite Load Caused by L. amazonensis

**Figure 4:**
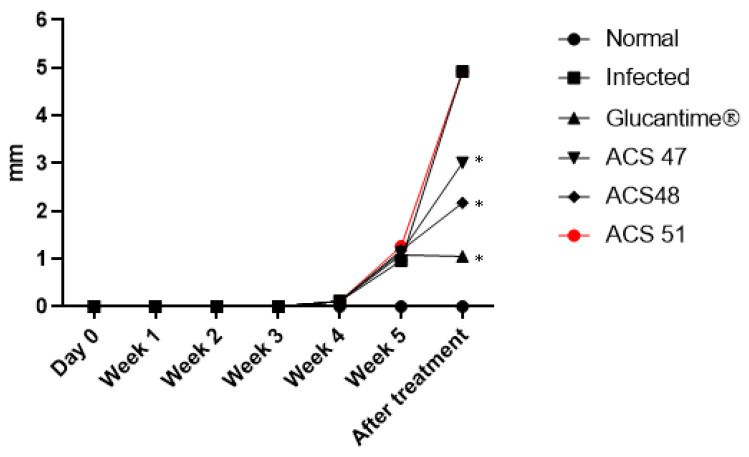
The size of the paw lesions and the curves demonstrate the evolution of the lesions on the right paws of the hamsters. Data represent paw thickness (mm) from two independent experiments with *n* = 4 per treatment. Measurements were taken for 5 weeks and 15 days after treatment. Statistical differences were performed using non-parametric Kruskal–Wallis and Mann–Whitney tests. (*) *p* < 0.05.

Lesion thickness in the infected right paws was measured using a Mitutoyo^®^ caliper, and values from the left paws were subtracted as baseline controls. The Infected and ACS51 groups exhibited the largest lesions (4.92 mm), with no significant differences between them. The Glucantime^®^ group showed the smallest lesions (1.05 mm), followed by the ACS48 group (2.17 mm). ACS47 also reduced lesion thickness (3.01 mm) (Figure 4).

**Figure 5:**
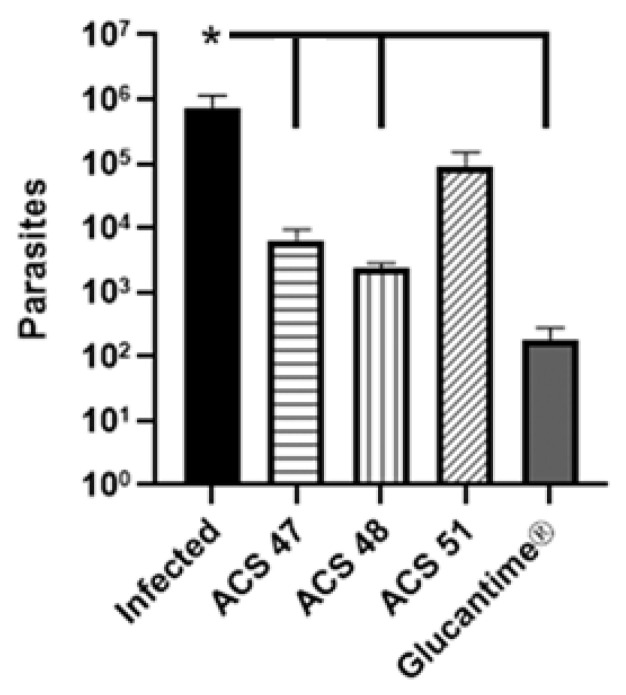
Evaluation of parasite load reduction in infected right paws. Parasite load was determined by limiting dilution of whole paws. Two independent experiments were conducted with *n* = 4 per treatment. (*) *p* < 0.05.

The LDA results corroborated those of the lesion-size evaluation. ACS47, ACS48, and Glucantime^®^ reduced parasite load by 99.1%, 99.7%, and 99.9%, respectively, in comparison to infected and untreated group (Figure 5).

### 2.4. Evaluation of Renal and Hepatic Toxicity Induced by the Treatments

**Figure 6:**
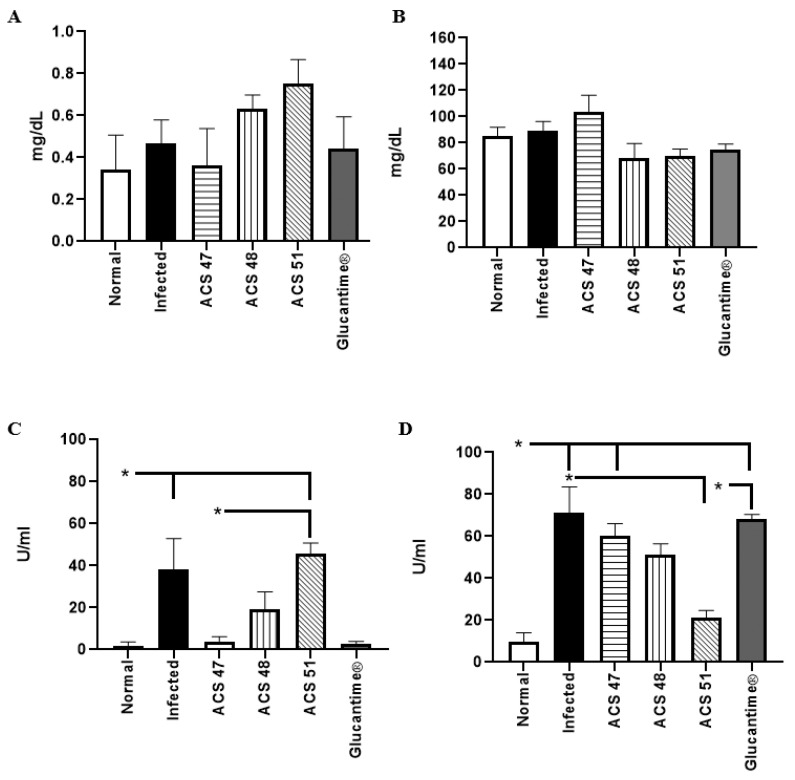
Evaluation of renal and hepatic toxicity markers after treatment of lesions caused by *L. amazonensis*. (**A**). Creatinine. (**B**). Urea. (**C**). ALT. (**D**). AST. Two independent experiments with *n* = 4 hamsters per group were performed. (*) *p* < 0.05.

Serum creatinine, urea, ALT, and AST levels were evaluated after completion of treatment. Renal function remained preserved in all treatments, with no significant differences compared to the normal group (*p* > 0.05) (Figure 6A,B). In contrast, alterations were observed in liver function markers. ALT levels (Figure 6C) increased in the Infected group and remained elevated in the group treated with ACS51 (*p* < 0.05), whereas treatment with ACS47, ACS48, and Glucantime^®^ reduced ALT levels by 89.6%, 49.9%, and 93.3%, respectively. AST levels were highest in the Glucantime^®^ and Infected groups compared with the Normal control group (*p* < 0.05), while ACS51 treatment reduced AST levels by 70.0% (Figure 6D).

### 2.5. Histology

**Figure 7:**
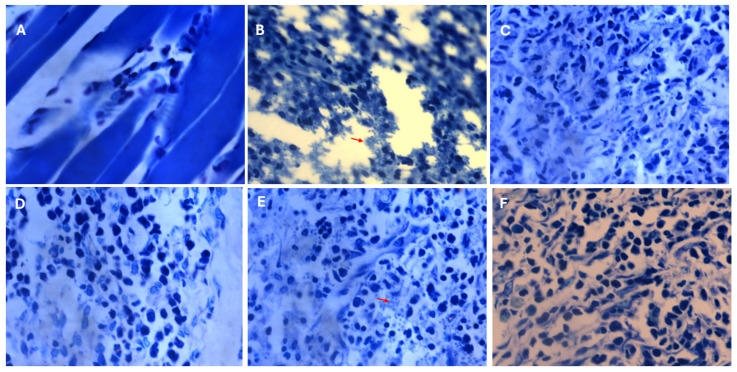
Tissue from paws infected with *L. amazonensis* and stained with hematoxylin and eosin (HE). All images were acquired using a 100× objective. (**A**). Normal, uninfected animal. (**B**). Infected group. (**C**). Treatment with ACS47. (**D**). Treatment with ACS48. (**E**). Treatment with ACS51. (**F**). Treatment with Glucantime^®^.

Histological analysis of paw plantar tissues was performed to visualize amastigote forms resulting from experimental infection. The infected group (Figure 7B) presented unstructured tissue with empty spaces and numerous amastigotes, indicating severe tissue infection. In contrast, tissues from the ACS47, ACS48, and Glucantime^®^ groups (Figure 7C,D,F) exhibited preserved structure with very few visible amastigotes. Conversely, the ACS51-treated group (Figure 7E) displayed numerous amastigotes and tissue characteristics similar to those observed in the Infected group.

### 2.6. In Silico Analysis

#### 2.6.1. ADME Properties of ACS47, ACS48 and ACS51

The three coumarins displayed distinct yet convergent ADME signatures that support their progression as drug-like scaffolds against *Leishmania* targets, with ACS47 emerging as the most chemically lean analogue (MW 230.26 g·mol^−1^; 17 heavy atoms; Csp3 = 0.21; 3 rotors) while ACS48 and ACS51 progressively increased in structural complexity (MW 298.38 and 358.43 g·mol−1; 6 and 8 rotors; Csp3 = 0.32 and 0.38, respectively), which translated into predictable shifts in lipophilicity, polarity, and synthetic tractability (Supplementary Table S1). All ligands remained within Lipinski limits with no violations and preserved comparable bioavailability scores (0.55), high GI absorption, absence of P-gp efflux liability, and full compliance with Ghose, Veber, and Egan filters; however, ACS48 and ACS51 each showed a single Muegge violation linked to their higher hydrophobicity.

Log P (consensus) increased from ACS47 (3.03) to ACS48 (4.51) and ACS51 (4.42), concomitant with reduced ESOL-predicted aqueous solubility (−3.55, −4.84, −5.08, respectively), a trend consistent with their rising molar refractivity (67.73 to 104.28) and TPSA enlargement in ACS51 (57.90 Å2 vs. 39.44 Å2 for ACS47/ACS48). All three compounds were predicted to cross the blood–brain barrier, a consequence of low polarity and the absence of H-bond donors, although ACS51’s larger TPSA approached the upper permissive threshold and corresponded to slightly weaker predicted skin permeation (Log Kp = −4.81). Across CYP liabilities, ACS47 and ACS48 were dual inhibitors of CYP1A2 and CYP2C19, whereas ACS51 showed no predicted CYP inhibition, which may reduce metabolic interactions during combination therapy. None triggered PAINS alerts, though all carried two Brenk structural alerts intrinsic to the coumarin core and isolated alkene motifs, in line with expectations for this chemical class.

Hence, collectively, the ADME landscape positions ACS47 as the most soluble and metabolically interactive analogue, ACS48 as the most hydrophobic but still rule-compliant, and ACS51 as the largest and most polar, yet with the cleanest CYP profile, providing a spectrum of physicochemical and pharmacokinetic attributes compatible with downstream docking-MD prioritization and early drug-discovery filters for *Leishmania* enzymes.

#### 2.6.2. Interaction Profile of a Putative LaSpdSyn@coumarins Complex Based on Molecular Docking

**Figure 8:**
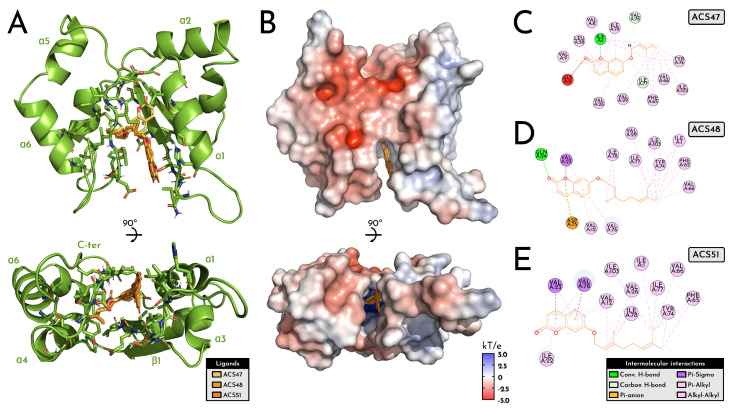
Structural and interaction landscape of *L. amazonensis* spermidine synthase bound to ACS47, ACS48, and ACS51. (**A**). The enzyme displays a compact α/β fold (six helices, α1–α6; two strands, β1–β2) enclosing a deep catalytic cleft. All three coumarins dock into the same hydrophobic site, positioning their coumarin rings toward the α1/β1 face and orienting their prenyl/geranyl chains toward the α4–α5 region. The 110-residue protein (~12.3 kDa) is enriched in hydrophobic and acidic residues, supporting a stable apolar pocket that maintains a conserved ligand orientation with variation only in tail depth. (**B**). Electrostatic mapping shows a neutral-to-acidic entrance (Asp/Glu) leading into a strongly hydrophobic trench dominated by Val, Ile, Leu, and Phe, creating an amphipathic gradient that guides the coumarin core inward and accommodates increasing substituent size (ACS47 < ACS48 < ACS51) without steric or electrostatic strain. (**C**). ACS47 is stabilized mainly by hydrophobic contacts across α1, β1, α4, and the α4–α5 loop, plus a single H-bond to I1 at the N-terminal loop; an unfavorable acceptor–acceptor contact with G2 is also present. The prenyl chain is enclosed by V66, F65, Y74, V76, I77, I7,8 and I103, forming a tight apolar cage around the ligand. (**D**). ACS48 engages a similar apolar tunnel but is anchored by two polar interactions: an H-bond with Q24 (loop α1–β1) and a π–anion interaction with D75 (loop α4–α5). Additional π–σ and alkyl/π-alkyl contacts with residues from α1, β1, α4, and the α4–α5 loop (notably V12, V23, V29, F65, V66, Y74, I77–I78, I103) define a well-distributed hydrophobic network along the ligand axis. (**E**). ACS51 interacts almost exclusively through hydrophobic and π-alkyl contacts at the cavity entrance and along the prenyl–geranyl chain, involving V12, V23, I22, V26, V76, I1, I77, I78, F65, V66 and Y74. This extended apolar set tightly frames both the coumarin core and the aliphatic tail, reflecting its deeper occupancy of the lipophilic cleft.

The putative *L. amazonensis* spermidine synthase (LaSpdSyn) model adopts a compact α/β scaffold with six α-helices and two β-strands (110 residues; ~12.3 kDa), enclosing a deeply recessed catalytic cavity that concentrates hydrophobic (≈50%) and acidic residues (Asp 9.09%; Glu 3.64%), consistent with a stable, apolar pocket suited for aromatic ligands (Figure 8). In all docking runs, ACS47, ACS48 and ACS51 converged to the same hydrophobic cleft, aligning their coumarin nuclei toward the α1/β1 face and projecting their prenyl or geranyl chains toward the α4–α5 region.

Electrostatic surface mapping showed a neutral-to-acidic entrance enriched in Asp/Glu that funnels the ligands into a strongly hydrophobic interior dominated by Val, Ile, Leu and Phe, in line with the amino acid composition (Val 13.64%, Gly 12.73%, Ile 8.18%, Ala 8.18%, acidic residues 12.7% total) and supporting accommodation of increasingly bulkier side chains (ACS47 < ACS48 < ACS51) without evident steric or electrostatic penalties (Figure 8). This amphipathic gradient, coupled with the deeply recessed trench spanning the N-terminal loop, α1, β1 and the α4–α5 loop, defines a continuous tunnel that overlaps the substrate-binding groove described for *Leishmania* spp. polyamine metabolism.

At the level of individual complexes, ACS47, ACS48, and ACS51 share a clear set of interaction hotspots, while differing in the number and distribution of polar anchors that fix the coumarin core in the cavity (Figure 8). ACS47 relies on a single directional H-bond between its ether oxygen and I1 (N-terminal loop), plus a dense alkyl/π-alkyl belt involving Val8 and Val9 (α1), Leu28 and Val29 (β1), and the α4/α4–α5 region (Phe65, Val66, Val76, Ile77, Ile78, Tyr74), with an unfavorable acceptor–acceptor proximity to Gly2, marking a local steric/electrostatic constraint at the mouth of the site. ACS48 strengthens this anchoring by adding a conventional H-bond with Gln24 (loop α1–β1) at the coumarin carbonyl and a π–anion contact with Asp75 (loop α4–α5), while preserving the same hydrophobic tunnel seen for ACS47, again centred on Val12 (α1), Val23/Val29 (loop α1–β1/β1), Val76/Ile77/Ile78 (loop α4–α5), Ile1 (N-terminal loop), Ile103 (C-terminal loop), and the α4 cluster Phe65/Val66/Tyr74.

In addition, ACS51, the most extended ligand, engages virtually the same hydrophobic scaffold but almost exclusively through alkyl/π-alkyl contacts, with the coumarin system contacting Val12 (α1), Val23 and Ile22 (loop α1–β1) and Val76, and the elongated prenyl–geranyl chain threading deeper along Val26 (β1), Ile77/Ile78 (loop α4–α5), Ile1 and again Phe65/Val66/Tyr74 at the distal end. The recurrence of N-terminal loop (I1), α1/β1 residues (Val12, Val23, Val26, Val29), and the α4–α5 cluster (Phe65, Val66, Tyr74, Val76, Ile77, Ile78, Ile103) across all three ligands indicates a conserved binding epitope that closely matches the substrate channel of LaSpdSyn, supporting a common mechanism in which coumarins plug the catalytic groove through a shared coumarin anchor plus a variable isoprenoid wedge, providing a structurally coherent explanation for their convergent docking and MD stability profiles and for their in vivo leishmanicidal activity (Figure 8).

#### 2.6.3. Molecular Dynamics and Structural Shifts of LaSpdSyn@coumarins Complex

Briefly, other initially promising targets, such as cytochrome c peroxidase (Q4Q3K2), aquaglyceroporin AQP1 (Q4Q6R2), cytochrome bc1 (P14548) and AdoMetDC (Q25264), were ultimately excluded after failing key validation steps in the pipeline, including loss of heme-cofactor engagement (e.g., in cytochrome-dependent enzymes), MD-driven instability/unbinding, or violation of predefined structural and energetic thresholds.

**Figure 9:**
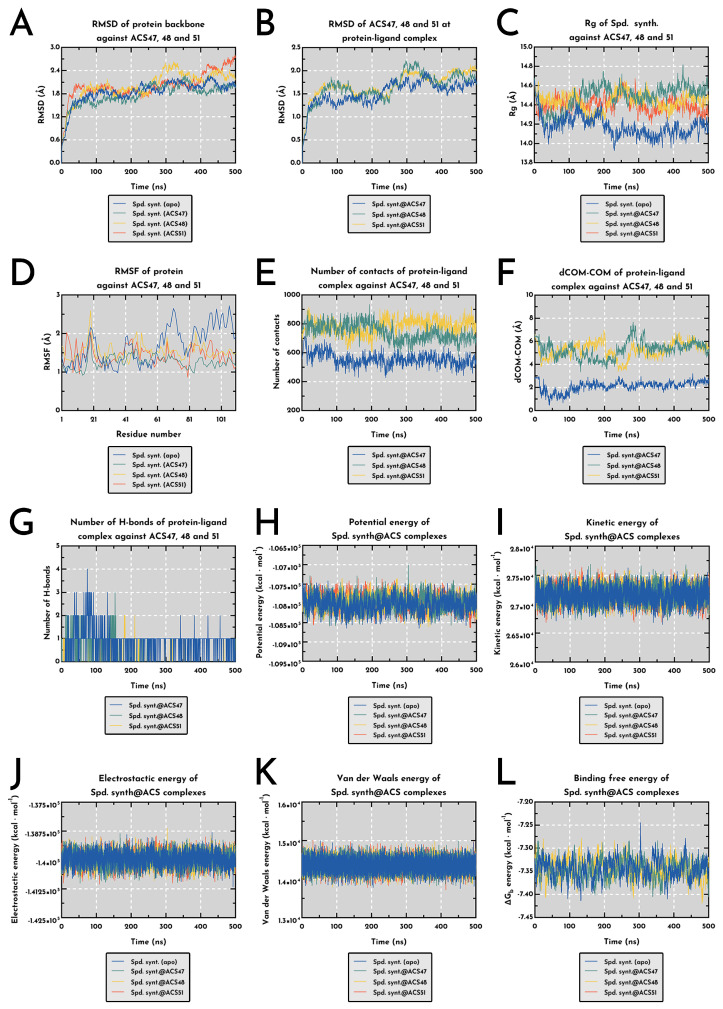
Structural and energetic behavior of a putative LaSpdSyn bound to active coumarins (ACS47, ACS48, and ACS51) along 500 ns MD simulations (n = 3). (**A**). Backbone RMSD showed the apo enzyme stabilizing at 1.85 ± 0.30 Å, while ACS47, ACS48 and ACS51 reached 1.77 ± 0.29, 2.01 ± 0.37 and 2.01 ± 0.34 Å (**** *p* < 0.0001), with ACS48/ACS51 drifting more than ACS47. (**B**). Ligand RMSD remained lowest for ACS47 (1.52 ± 0.26 Å), followed by ACS48 (1.65 ± 0.30 Å) and ACS51 (1.69 ± 0.27 Å) (**** *p* < 0.0001). (**C**). Rg indicated compaction upon binding, with apo at 14.18 ± 0.11 Å vs. 14.49 ± 0.12 Å (ACS47), 14.44 ± 0.09 Å (ACS48), and 14.40 ± 0.08 Å (ACS51) (**** *p* < 0.0001). (**D**). RMSF showed ACS47 dampening flexibility (1.20 ± 0.18 Å) relative to apo (1.43 ± 0.43 Å), ACS48 (1.29 ± 0.33 Å), and ACS51 (1.40 ± 0.23 Å) (**** *p* < 0.0001). (**E**). Contact density ranked ACS51 (783.8 ± 52.6) > ACS48 (742.5 ± 58.8) > ACS47 (556.7 ± 44.3) (**** *p* < 0.0001). (**F**). dCOM–COM distances split into two regimes: ACS47 remained close (2.10 ± 0.44 Å), while ACS48 (5.28 ± 0.71 Å) and ACS51 (5.25 ± 0.67 Å) adopted a more distant mode (**** *p* < 0.0001). (**G**). Hydrogen bonding followed ACS47 (0.86 ± 0.59) > ACS48 (0.29 ± 0.54) > ACS51 (0.06 ± 0.26) (**** *p* < 0.0001). (**H**). Potential energy differed modestly, with apo at –108 037 ± 183 kcal mol^−1^ vs. −107 966 ± 184 (ACS47), −107 981 ± 178 (ACS48), and −107 947 ± 178 (ACS51) (**** *p* < 0.0001). (**I**). Kinetic energy showed small shifts (124.5, **** *p* < 0.0001), with ACS47 slightly higher (27 177 ± 131 kcal mol^−1^). (**J**). Electrostatic terms decreased for ACS47 and ACS51 (−36.9 and −34.4 kcal mol^−1^; **** *p* < 0.0001), while ACS48 stayed comparable to apo. (**K**). van der Waals contributions varied minimally (6.56, *p* = 0.0002), with a significant shift only for apo vs. ACS51 (+9.90 kcal mol^−1^, **** *p* < 0.0001). (**L**). ΔGbind values clustered tightly (−7.344 to −7.346 kcal mol^−1^), yet showed subtle distinctions (36.45, **** *p* < 0.0001), ACS48 being marginally more stable than ACS47, with ACS51 slightly detached from both.

Hence, across the 500 ns trajectories, spermidine synthase displayed a consistent but ligand-dependent structural response, with ACS47 producing the most compact and internally coherent complex (Figure 9). Protein backbone RMSD stabilized near 1.85 ± 0.30 Å for the apo form and shifted to 1.77 ± 0.29 Å, 2.01 ± 0.37 Å, and 2.01 ± 0.34 Å for ACS47, ACS48, and ACS51, respectively (**** *p* < 0.0001), indicating that ACS47 maintained a tighter conformational basin, whereas ACS48/ACS51 induced sustained drift. Ligand RMSD profiles followed the same hierarchy, with ACS47 showing the lowest internal mobility (1.52 ± 0.26 Å) (**** *p* < 0.0001) and suggesting an energetically rooted docking pose that resisted long-timescale disruption. Binding markedly affected global compaction: the radius of gyration contracted relative to the apo condition and remained stable for all complexes, more prominently with ACS47 (14.49 ± 0.12 Å) (**** *p* < 0.0001), supporting ligand-induced tightening of the core (Figure 9B,C). Local dynamics (RMSF) confirmed a damping effect driven predominantly by ACS47 (1.20 ± 0.18 Å), which significantly reduced mobility in flexible loops compared with apo (1.43 ± 0.43 Å) and with ACS48 (1.29 ± 0.33 Å) (**** *p* < 0.0001) (Figure 9D).

Additionally, contact analyses reinforced this interpretation but revealed distinct mechanistic regimes: ACS51 generated the densest contact network (783.8 ± 52.6), followed by ACS48 (742.5 ± 58.8) and ACS47 (556.7 ± 44.3) (**** *p* < 0.0001), indicating that ACS48/ACS51 rely on broader packing rather than precise anchoring. This was mirrored by dCOM–COM separations, where ACS47 remained consistently proximal to the catalytic pocket (2.10 ± 0.44 Å), whereas ACS48 and ACS51 occupied a more distant mode (~5.26 Å; **** *p* < 0.0001). Hydrogen bonding provided the clearest discriminator: ACS47 maintained the highest count (0.86 ± 0.59), far surpassing ACS48 (0.29 ± 0.54) and ACS51 (0.06 ± 0.26) (**** *p* < 0.0001). Together, these metrics show that ACS47, unlike the more diffusely accommodated ACS48/ACS51, stabilizes the catalytic domain through persistent, short-range interactions (Figure 9E,F).

Energy decomposition further supported the existence of two mechanistic classes and aligned with the structural evidence. Potential energy shifted moderately across complexes (**** *p* < 0.0001), with ACS51 producing the largest deviation from apo (−89.8 kcal mol^−1^), although not accompanied by stronger geometric stabilization. Kinetic energy differed slightly among groups (**** *p* < 0.0001), with ACS47 maintaining marginally higher values (27 177 ± 131 kcal⋅mol^−1^) that remained compatible with stable local minima along the MD landscape (Figure 9H,I). Electrostatic contributions favored ACS47 and ACS51 (mean shifts −36.9 and −34.4 kcal⋅mol^−1^ relative to apo; **** *p* < 0.0001), whereas ACS48 presented an electrostatic profile nearly identical to the unbound enzyme. Also, the van der Waals energies showed only minor divergence, with ACS51 producing the sole significant enhancement (+9.90 kcal mol^−1^; **** *p* < 0.0001) (Figure 9J,K).

Despite the divergent interaction patterns, the calculated binding free energies remained tightly distributed (−7.344 to −7.346 kcal⋅mol^−1^; **** *p* < 0.0001), with ACS48 marginally more favorable than ACS47 (**** *p* < 0.0001) (Figure 9L). When considered together, these structural and energetic signatures indicate that these coumarins exert a coherent and plausible inhibitory effect on *L. amazonensis* spermidine synthase, albeit through distinct binding behaviors: ACS47 stabilizes the catalytic environment through precise, hydrogen-bonded anchoring, whereas ACS48 and ACS51 rely on broader contact surfaces and electrostatic/dispersion complementarity.

#### 2.6.4. SpdSyn Conservancy on the *Leishmania* Genus Based on Sequence and Structure Alignment

**Figure 10:**
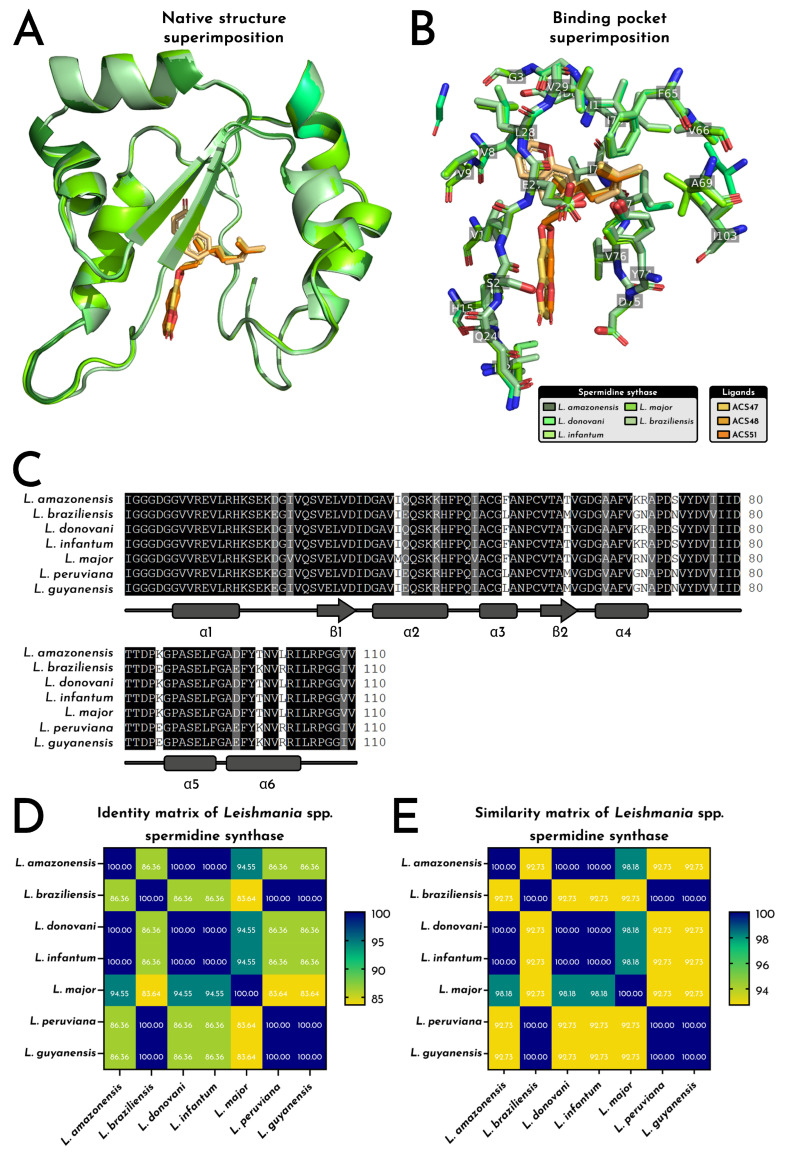
Structural and sequence conservation of *Leishmania* spp. spermidine synthase and binding-site convergence relevant for ACS47, ACS48 and ACS51 interactions. (**A**). Native structure superimposition. Superimposition of spermidine synthase models from *L. amazonensis*, *L. braziliensis*, *L. guyanensis*, *L. donovani*, *L. infantum*, *L. major* and *L. peruviana* reveals a highly conserved fold, with backbone RMSD values against *L. amazonensis* ranging from 0.112 Å (*L. infantum*) to 0.366 Å (*L. braziliensis*), supporting a stable structural framework for ligand accommodation. The docked coumarins (ACS47, ACS48, and ACS51) occupy overlapping regions within the canonical catalytic cavity. (**B**). Binding-pocket superimposition. Local alignment of catalytic residues shows strong conservation of side-chain orientation across species, with minimal positional drift in key residues (e.g., V29, E42, V76, F65). (**C**). Multiple-sequence alignment. Spermidine synthase sequences exhibit extensive conservation across species, with long blocks of invariant residues spanning α1–α6 and β1–β2. Pairwise identities range from 83.64% (*L. braziliensis* vs. *L. major*) to 100% (*L. donovani* vs. *L. infantum*), with similarity consistently high (92.73–100%). Such conservation supports the near-identical tertiary organization observed in panels (**A**,**B**). (**D**). Identity matrix of *Leishmania* spp. spermidine synthase. Matrix representation confirms the narrow identity distribution (≈84–100%), with the closest cluster formed by *L. donovani, L. infantum*, and *L. peruviana* (100%), while *L. amazonensis* maintains ≥ 94.55% identity with most species and 86.36% with *L. braziliensis*. (**E**). Similarity matrix of *Leishmania* spp. spermidine synthase. Similarity indices remain uniformly high (92.73–100%), further highlighting evolutionary conservation of both sequence chemistry and functional motifs.

Structural comparison of a putative LaSpdSyn demonstrated that the *L. amazonensis* enzyme retains a highly conserved α/β architecture (six α-helices, two β-strands) across the genus, with backbone RMSD values remaining below 0.40 Å in all pairwise superimpositions (0.112–0.366 Å), indicating negligible deviations in global fold and no distortions within the catalytic chamber (Figure 10A). Binding-site alignment confirmed that the catalytic pocket is conserved in both sequence and side-chain orientation, with residues lining the cavity (e.g., Val29, Glu42, Val76, Phe65, Gln24, Met45) maintaining identical rotameric dispositions across species and showing no steric clashes against ACS47, ACS48, or ACS51 during docking or MD refinement (Figure 10B).

Also, multiple-sequence alignment revealed long invariant blocks spanning α1–α6 and β1–β2, consistent with the tightly clustered identity (83.64–100%) and similarity (92.73–100%) matrices (Figure 10C–E). Notably, *L. amazonensis* shared ≥94.55% identity with *L. major*, *L. donovani*, *L. infantum*, and *L. peruviana*, with the lowest but still high value registered against L. braziliensis (86.36%), while similarity remained uniformly elevated (92.73–100%), reinforcing the evolutionary stability of catalytic motifs essential for polyamine biosynthesis. These structural and sequence constraints explain the uniform ligand accommodation observed for ACS47, ACS48, and ACS51, whose docked poses overlapped without requiring side-chain rearrangements, supporting a shared mechanism of inhibition of a deeply conserved enzymatic target across *Leishmania* spp. and strengthening the biological plausibility of spermidine synthase as the operational target underlying the in vivo efficacy of these coumarins.

## 3. Discussion

Leishmaniasis remains therapeutically challenging due to the toxicity and limited efficacy of current drugs, reinforcing the need for safer and more effective alternatives [16]. Previously, our group synthesized altissimacoumarin D (ACS51) and related derivatives and demonstrated their antifungal activity [15]. Additionally, auraptene (ACS48) has been reported to display activity against *L. major*. These findings provided a rationale to investigate whether structural variations within 7-prenyloxycoumarins could confer activity against *L. amazonensis*.

Structure–activity analysis revealed that substitution pattern and side-chain length markedly influenced antileishmanial activity. The complete loss of activity observed for ACS56, together with the weak performance of its structural analogue ACS55, suggests that extensive methyl substitution at the coumarin core negatively impacts biological activity, likely due to steric hindrance affecting target engagement or membrane interaction. Compounds bearing fewer methyl substituents displayed improved activity, reinforcing the relevance of steric balance within the scaffold. Among the most active derivatives, geranylated coumarins outperformed their prenylated counterparts, maintaining activity at lower concentrations. This trend is consistent with previous reports describing the antileishmanial effect of auraptene (ACS48) against *L. major* [17], although the lower susceptibility observed for *L. amazonensis* suggests potential species-dependent differences. The enhanced performance of geranylated analogues may reflect increased lipophilicity and improved interaction with hydrophobic enzymatic pockets [18].

To further assess selectivity, the most active derivatives were evaluated for cytotoxicity in HaCaT keratinocytes, a relevant model given the cutaneous nature of *L. amazonensis* infection. Overall, the compounds demonstrated a favorable selectivity profile, particularly ACS47, which exhibited the lowest cytotoxicity. Although ACS48 and ACS51 showed moderate effects on keratinocyte viability, their antiparasitic activity remained proportionally higher, indicating preferential toxicity toward the parasite. These findings align with previous evidence from our group showing absence of hemolytic activity and with in silico toxicity predictions suggesting low mutagenic, tumorigenic, or reproductive risk [15]. Together, the data support a preliminary safety profile compatible with further pharmacological development.

Previous toxicological evaluation of ACS48 in rodents demonstrated a favorable safety profile at substantially higher doses than those employed in this study [19], supporting its progression to in vivo assessment. In the hamster model, the active coumarins significantly reduced parasite burden, confirming their biological efficacy in a mammalian system. Notably, ACS51, although the most potent compound in vitro, displayed reduced in vivo performance. This discrepancy may reflect metabolic liability, as its methoxy substituents could undergo CYP450-mediated O-demethylation, potentially generating less active metabolites [20]. Consistent with parasitological findings, ACS47 and ACS48 also promoted measurable lesion improvement, whereas ACS51 failed to induce visible clinical regression. Considering the strong inflammatory component associated with *L. amazonensis* lesions [21], differential modulation of host inflammatory responses may also contribute to these outcomes.

In addition to evaluating therapeutic efficacy, systemic safety was investigated to determine whether the observed antiparasitic effects were accompanied by organ toxicity. Biochemical markers indicated preserved renal function across all groups, reinforcing the absence of nephrotoxicity, a relevant finding given the well-documented renal limitations of amphotericin B. In contrast, infection was associated with elevated hepatic transaminases, consistent with liver involvement during *L. amazonensis* infection [22]. Treatment with ACS47 and ACS48 normalized ALT levels without affecting AST, suggesting not only a lack of hepatotoxicity but also a possible attenuation of infection-induced hepatic alterations. The previously reported hepatoprotective properties of ACS48, mediated through activation of the Keap1/Nrf2 antioxidant pathway [23], may contribute to this effect. Coumarin derivatives are historically associated with anticoagulant activity, but no hemorrhagic events were observed in treated animals during the in vivo experiments, indicating no clinically evident anticoagulant effects under the tested conditions. These findings indicate a favorable systemic safety profile for the leading compounds. A limitation of the present study is the modest group size, which may limit statistical power in pairwise subgroup comparisons. Post hoc power analysis indicated high statistical power for endpoints showing large effects, whereas parameters with smaller effects presented only moderate power. Therefore, non-significant differences in some comparisons should be interpreted with caution and cannot be considered definitive evidence of biological equivalence.

To elucidate the mechanism underlying the antiparasitic activity of ACS47, ACS48, and ACS51, integrated in silico analyses were performed, converging on spermidine synthase (LaSpdSyn) as a plausible molecular target. ADME profiling positioned the three coumarins within a drug-like envelope compatible with oral exposure and macrophage uptake, while revealing a progressive increase in lipophilicity (ACS47 < ACS48 < ACS51) that is mechanistically relevant for interaction with the deeply recessed, hydrophobic catalytic groove of SpdSyn [24,25]. This pattern aligns with polyamine-pathway inhibitors described for trypanosomatids, including geraniol/linalool analogues and scaffolds derived from large virtual screens, which typically exploit hydrophobic complementarity within the putrescine channel and dcSAM trench while maintaining limited polarity to avoid solvent-driven destabilization [26,27,28,29,30,31,32]. Notably, ACS47’s lower lipophilicity and preserved directional H-bonding align with gatekeeping–stabilizing ligands, whereas ACS48/ACS51 show the hydrophobic packing typical of vdW-driven stabilization reported for monoterpenoid and other coumarin-like inhibitors [26,27,33].

The molecular docking and MD analyses reinforce a mechanistic paradigm consistent with established studies of SpdSyn inhibition: ligands that exert the strongest perturbation of catalytic dynamics are those that engage both the putrescine channel and the conserved gatekeeping loop spanning the Asp/Gln/Thr motif, a region known to control substrate access and product release in *Leishmania* and *Trypanosoma* spp. [26,27,34]. The persistent interactions identified for ACS47, particularly those stabilizing the α1–β1 interface and restricting loop mobility, mirror the behavior of Lig1/Lig2 identified by [29], which reduced conformational freedom of LdSpdSyn through specific H-bond pinning at Asp123/Asp176. Conversely, the contact-dense but less geometrically restrictive modes adopted by ACS48/ACS51 resemble the vdW-dominated regimes proposed for GER/LIN, which achieved stability not through discrete H-bonds but via distributed hydrophobic engagement of residues analogous to Val62, Ile63, Tyr73, Tyr243, and Ile248 in LdSpdSyn [27,35].

In our simulations, ACS48/ACS51 similarly occupied the cavity through extensive apolar networks in the α4–α5 region, reproducing the mechanistic signatures of ligands that stabilize the enzyme through packing rather than pinning [27]. Importantly, all three coumarins preserved compatibility with the catalytic lining without forcing side-chain displacement, a hallmark of true SpdSyn binders noted across comparative docking/MD studies of putrescine analogues, monoterpenoids, and dcSAM-mimics [26,36]. Also, the remarkably tight ΔGbind distribution across ACS47/48/51 supports a binding mechanism that, while differentiated in its local interaction chemistry, converges energetically toward functionally relevant inhibition.

The conservation analysis further supports spermidine synthase as the operative target underlying the in vivo activity of these coumarins. The preserved α/β scaffold, dcSAM trench, and putrescine-binding architecture across *Leishmania* spp. align with genetic evidence showing that SpdSyn disruption compromises infectivity and downstream trypanothione and hypusine biosynthesis [26,27]. The structural invariance of the gatekeeping loop also explains the cross-species susceptibility of inhibitors targeting this microenvironment [33]. Mechanistically, ACS47 displayed features consistent with gatekeeper-restricting inhibitors that limit catalytic flexibility [37,38]. Meanwhile, the broader but stable engagement observed for ACS48/ACS51 is consistent with ligands that occupy the putrescine channel in non-canonical extended poses, similar to the hydrophobic monoterpenoids that outperform the natural substrate in vdW energy contributions [26,27,35].

Taken together, data indicate that the coumarins engage a structurally conserved SpdSyn catalytic chamber and perturb conformational elements essential to polyamine biosynthesis, in line with established inhibitors of this pathway [26,27,35]. Also, inhibition of the polyamine pathway has been demonstrated to abruptly halt intermediates production, collapsing trypanothione biosynthesis and thereby dismantling redox homeostasis, DNA replication, and mitochondrial integrity. Therefore, its disruption is expected to compromise parasite viability [28,30]. Accordingly, the putative LaSpdSyn emerges as a biologically and structurally substantiated target underlying the antiparasitic activity of ACS47, ACS48, and ACS51. Within this context, the relevance of these findings extends beyond mechanistic understanding. Cutaneous leishmaniasis remains highly prevalent in endemic regions, where therapies are limited by toxicity, parenteral administration, and prolonged treatment regimens. The identification of compounds displaying in vivo efficacy and a favorable systemic safety profile supports the investigation of 7-prenyloxycoumarins as alternative therapeutic candidates. Although further pharmacokinetic and translational studies are required, these results may contribute to the development of safer and more accessible treatments and thereby help strengthen the antileishmanial control pipeline.

## 4. Materials and Methods

### 4.1. Compounds

**Figure 11:**
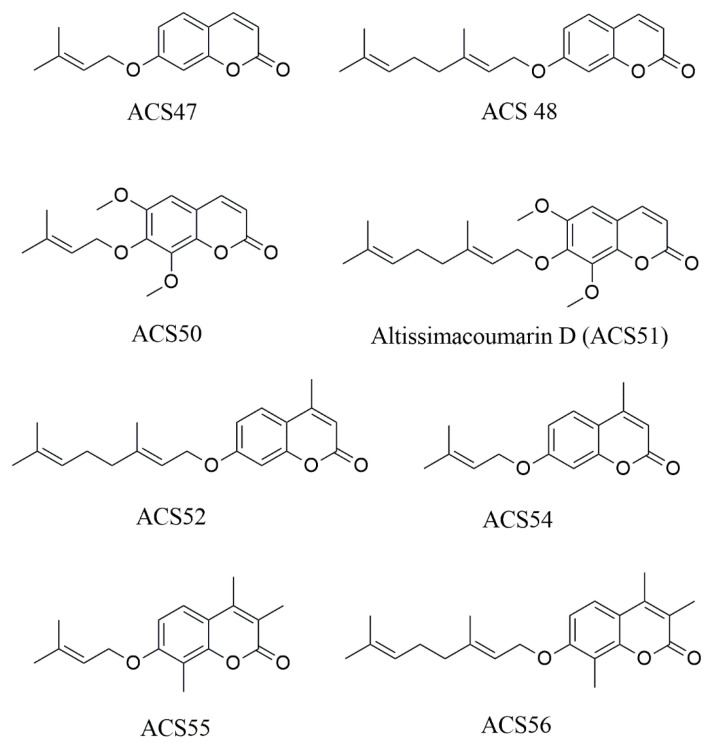
Chemical structure of the 7-prenyloxycoumarins tested in this study.

The prenylated coumarins (Figure 11) were synthesized as described by Silva et al., 2023 [15], diluted in dimethyl sulfoxide (DMSO; Sigma-Aldrich, St. Louis, MO, USA) to 10 mg/mL, and maintained at room temperature.

### 4.2. Parasites

Promastigote forms of *Leishmania amazonensis* (IFLA/BR/1967/PH8) from the *Leishmania* Collection of the Oswaldo Cruz Institute (CLIOC/FIOCRUZ/RJ) were maintained at 26 °C in Schneider culture medium (Sigma-Aldrich, St. Louis, MO, USA) supplemented with 10% fetal bovine serum (FBS; Cultilab, Campinas, São Paulo, Brazil) and subcultured weekly until the tenth passage. For infection of the animals involved in the in vivo experiments, infective metacyclic promastigote forms were obtained by culturing macerated infected paws in Schneider medium supplemented with 10% FBS. The macerated paws were added to sterile 24-well plates and serially diluted to the end of the wells. The plates were incubated at 26 °C and observed daily under a microscope until the promastigote forms were fully differentiated. Then, the promastigote forms were transferred to 25 cm^2^ tissue culture flasks containing 10 mL of Schneider medium supplemented with FBS and 10% *Leishmania* inoculum. Finally, the cultures were expanded to 10^6^ promastigotes/mL.

### 4.3. Evaluation of Coumarins’ Effects on Promastigote Viability

The effect of the compounds on promastigote viability was measured through a microdilution assay [39]. Firstly, a screening assay was performed. Promastigotes in exponential growth phase (1 × 10^6^ cells/mL) were incubated in Schneider medium supplemented with 10% FBS, in the presence of coumarins at 100 μg/mL for 72 h at 26 °C. Afterwards, promastigote viability was quantified by the MTT ((3-(4,5-dimethylthiazol-2-yl)-2,5-diphenyltetrazolium bromide; Sigma-Aldrich, St. Louis, MO, USA) reduction assay. Briefly, cells were incubated with 5 mg/mL MTT at 37 °C for 3 h in the dark. Then, DMSO was added for 1 h at room temperature to dissolve the formazan crystals. The metabolic activity of cells was measured at 492 nm (Fluostar Optima, BMG Labtech, Offenburg, Germany), and results were expressed as percentage in comparison to untreated cells. Compounds that impaired *Leishmania* viability by >70% were then tested at serial concentrations (100–3.125 μg/mL), in the same conditions described above, and untreated cells were used as a positive viability control.

### 4.4. In Vitro Toxicity Against HaCaT Cells

The toxicity of the prenylated coumarins was assessed against human keratinocytes, using a microdilution assay with slight modifications [39]. Briefly, cells (2 × 10^5^/mL) were seeded into the wells of a 96-well polystyrene microplate and incubated at 5% CO_2_ atmosphere in Dulbecco’s Modified Eagle Medium (DMEM, Sigma-Aldrich, St. Louis, MO, USA) supplemented with 10% FBS, in the presence of coumarins ranging from 100 to 3.125 μg/mL for 48 h at 37 °C. Then, cell viability was evaluated using the neutral red assay.

### 4.5. Ethical Statement

For the in vivo experiments, Syrian hamsters were used in accordance with the ethics committee CEUA/CCS/UFRJ/Brazil protocol number 015/20. The hamsters were kept in microisolators at 24 °C and on a 12 h light/dark cycle. Food and water were offered ad libitum. All animals were observed daily and at the end of the experiment were euthanized with lethal doses of ketamine (300 mg/kg) and xylazine (30 mg/kg).

### 4.6. In Vivo Experimental Design

Two in vivo experiments (*n* = 4) were conducted using the hamster model, which is the best model for leishmaniasis because it mimics the disease’s evolution in humans. Female hamsters aged 2 months were infected by inoculation of 1 × 10^5^ cells/0.05 mL promastigote forms of *L. amazonensis* or PBS (uninfected control) through inoculation in the right hind paw. The animals were randomly placed in cages and evaluated daily to assess their health and weekly to measure paw thickness using a caliper (Mitutoyo, Kawasaki, Japan). In the fifth week, treatment was initiated and continued daily for 15 days. The experimental groups in this study were: Normal, Infected, Infected and treated with ACS 47, Infected and treated with ACS 48, Infected and treated with ACS 51, and Infected and treated with Glucantime^®^ (Sanofi, Paris, France). All drugs were administered at a concentration of 2.5 mg/kg via intraperitoneal injection. The day after the last dose, the thickness of the animals’ paws was measured again, and the animals were euthanized according to institutional policies. Subsequently, the paws containing the lesions were removed for parasite load analysis by limiting dilution assay (LDA) in 24-well plates. Blood from all animals was collected by cardiac puncture using an insulin syringe and transferred to 1.5 mL tubes. The blood samples were incubated for one hour at 37 °C. Next, they were centrifuged at 3000 rpm for 5 min, and the supernatant was collected and stored at −80 °C for subsequent measurement of urea, creatinine, alanine aminotransferase (ALT), and aspartate aminotransferase (AST). Muscle tissue from the infected paw was surgically removed for histological analysis [39].

### 4.7. Limiting Dilution Assay

The paws were removed after euthanasia and aseptically macerated in a laminar flow hood. For each paw, 1 mL of Schneider medium supplemented with 10% fetal bovine serum was used. The suspension was added to sterile 24-well plates, and serial dilutions were performed through the last well. The plates were then incubated at 26 °C for 7 days. The material was evaluated daily by optical microscopy until complete differentiation into promastigotes was visualized. The titer dilution was given by the last well containing *Leishmania* [39].

### 4.8. Evaluation of Renal and Hepatic Toxicity

The dosages were performed using commercial kits from Labtest (Lagoa Santa, MG, Brazil) according to the manufacturer’s instructions, adapting the reagent volume proportionally to the minimum sample volume recommended in the procedure protocol. For the evaluation of renal function, urea and creatinine levels were monitored, and for the assessment of hepatic function, ALT and AST levels were measured in the serum after complete treatment with the drugs involved in the experiment [39].

### 4.9. Analysis of Histological Sections

After euthanasia of the animals, the tissue from the paws was removed and immediately immersed in 4% paraformaldehyde (Sigma Aldrich, St. Louis, MO, USA) in phosphate buffer. After 24 h, the material was dehydrated following a progressive increase in ethanol concentration: 30 min in 70% ethanol (Merck, Darmstadt, Germany), 30 min in 80% ethanol, 30 min in 90% ethanol, and 30 min in 100% ethanol (repeated twice). Next, the samples underwent clarification, followed by two consecutive 30 min incubations in xylene (Merck, Darmstadt, Germany). The next step was to impregnate the material with paraffin. Two paraffin baths were performed for 30 min at 60 °C. Finally, the samples were embedded in plastic cassettes for histological sections. The sections were cut at a thickness of 5 µM using a microtome in the multi-user histology lab of the Biophysics Institute. Microscope slides were previously treated with poly-L-lysine (Sigma Aldrich, St. Louis, MO, USA), dried at room temperature, and the histological sections were placed on the slides. The slides were stained with hematoxylin and eosin. The samples were analyzed and the images acquired using the Axioplan 2 microscope (100×) at the Microscopy Unit of the Paulo de Góes Institute of Microbiology [40].

### 4.10. Statistical Analysis for In Vitro and In Vivo Experiments

The statistical analyses were performed using the nonparametric Kruskal–Wallis and Mann–Whitney tests (GraphPad Prism 9). An effect-size and post hoc power assessment were performed for the omnibus group comparisons. For the Kruskal–Wallis analyses we report epsilon-squared (ε^2^ = (H − k + 1)/(n − k)) as a rank-based estimate of the magnitude of group differences, and we provide an approximate achieved power for the omnibus effect (α = 0.05) using an ANOVA-equivalent noncentral-F approximation.

### 4.11. In Silico Analysis

#### 4.11.1. ADME Properties of ACS47, ACS48 and ACS51

The coumarins were manually reconstructed in ChemDraw 3D Pro v12.0.2 (Revvity Signals Software, USA), and their curated structures were subsequently submitted to SwissADME for physicochemical and pharmacokinetic evaluation [41,42,43].

#### 4.11.2. Molecular Docking Evaluation of *L. amazonensis* Strain PH8 Druggable Targets

In brief, all *L. amazonensis* strain PH8 protein targets emerging from the preliminary filtering step, namely enzymes with evidence of essentiality in core survival pathways and other processes critical to parasite cell biology, were retrieved from structural repositories (PDB, UniProt, and AlphaFold), encompassing both experimentally resolved structures and homology-based models [44,45]. A detailed target screening and docking protocol is described in the Supplementary Material; however, a concise synthesis is that the target screening pipeline consistently highlighted spermidine synthase as one of the metabolically relevant and structurally coherent targets for deeper investigation.

Hence, ACS47, ACS48, and ACS51 were docked into the active site of *L. amazonensis* spermidine synthase (LaSpdSyn) using AutoDock Vina 1.2.0 [46,47]. Protonation and tautomeric states for both receptor and ligands were first assigned for physiological pH 7.4, based on pKa estimates, before any coordinate conversions. Missing hydrogens were added consistent with pH 7.4 and Gasteiger charges were assigned, and the receptor was exported to .pdbqt with a rigid backbone and side chains.

Ligand preparation followed a standardized workflow. The 3D structures of ACS47, ACS48, and ACS51 were sketched in ChemDraw 3D Pro v12.0.2 (Revvity Signals Software, USA). Structures were energy-regularized using MM2 to an RMS gradient of 0.010 kcal·mol^−1^·Å^−1^ [48] and saved as .sdf or .mol2. All ligands were then protonated for pH 7.4, had polar hydrogens explicitly added, Gasteiger charges assigned, and were converted to .pdbqt; nonpolar hydrogens were left unmerged, and lone-pair modeling was enabled to preserve coumarin heteroatom and resonance chemistry [49,50].

The docking grid was centered on the dcSAM-binding cavity and catalytic His/Asp dyad described for trypanosomatid SpdSyn enzymes, encompassing the full substrate-access channel to permit accommodation of the bulky prenylated coumarins [26,29]. Grid parameters included: (i) exhaustiveness = 8; (ii) 10 output binding modes; and (iii) a maximum energy difference of 3 kcal·mol^−1^. For each ligand, the five top-ranked poses and any alternative poses within ~1.0 kcal·mol^−1^ of the best-scoring conformation were retained. Visual inspection in UCSF Chimera (version 1.18), PyMOL Molecular Graphics System (version 3.1, Schrödinger LLC) [51], and BIOVIA Discovery Studio v. 2024 [52] ensured sensible placement within the catalytic pocket, including: (i) hydrogen-bond geometry toward residues lining the dcSAM-binding region; and (ii) realistically oriented π–π or alkyl contacts with hydrophobic residues surrounding the adenine-binding subsite [53,54].

Finally, the best-scoring pose of each coumarin was exported from .pdbqt and converted to .mol2 via Open Babel 3.1.1 [55] for subsequent molecular dynamics parameterization.

#### 4.11.3. Molecular Dynamics Evaluation of *L. amazonensis* Strain PH8 Druggable Targets

All-atom molecular dynamics (MD) simulations were carried out to examine the structural stability and interaction profile of the *Leishmania amazonensis* spermidine synthase (LaSpdSyn) in complex with the three coumarins that showed the strongest in vivo and in silico performance (ACS47, ACS48, and ACS51). Simulations were performed in NAMD 3.0.1 with CUDA acceleration [56,57]. Protein atoms were described using the CHARMM36m force field, whereas ligand parameters were obtained from the CHARMM General Force Field (CGenFF) [58] and refined through the Force Field Toolkit (ffTK) implemented in VMD 1.9.4 [59,60,61]. Complex preparation followed a standard PSFGEN workflow, generating the full topology and coordinate set for each LaSpdSyn–coumarin system. Each complex was either placed in a TIP3P explicit solvent box under periodic boundary conditions and neutralized with Na^+^ and Cl^−^ ions to a physiological concentration of 0.15 M or inserted in a mixed lipid membrane model provided by CHARMM-GUI [62,63], depending on their role related to cell biology [26,64,65].

The MD protocol consisted of three main stages: energy minimization, equilibration, and production dynamics. Energy minimization was performed for 10,000 steps using the conjugate-gradient method to remove steric clashes and stabilize the LaSpdSyn–ligand interfaces. Equilibration followed a progressive heating schedule from 0 K to 310 K, increasing in 20 K increments every 10,000 steps. Temperature control was achieved using a Langevin thermostat (damping coefficient 1 ps^−1^) [66,67], and pressure was regulated when needed by a Langevin piston approach. A 1 fs timestep was used during minimization and equilibration to ensure stable relaxation of the active-site catalytic residues (i.e., the Asp–Gly–Asp motif and the polyamine-binding channel) [27,29]. Production simulations were extended to 500 ns for each complex (5 × 10^8^ steps at 1 fs). Non-bonded interactions used a 10 Å cutoff with a switching function starting at 9 Å and a 12 Å pair-list distance. Long-range electrostatics were computed using Particle Mesh Ewald (PME) [68,69].

Trajectories were saved every 1000 steps (.dcd), and energy logs were written with the same frequency (.log). Quantitative analyses were performed with MDAnalysis v. 2.9.0 [70], NumPy v.2.3.3 [71], Pandas v.2.3.3 [72], BioPython v.1.86 [73], and Matplotlib v.3.10.7 [74] at Python 3.14. Structural dynamics were evaluated through backbone and heavy-atom RMSD, per-residue RMSF, radius of gyration (Rg), hydrogen-bond occurrence and occupancy, center-of-mass distances (dCOM-COM), and the formation/loss of contacts within ≤4.5 Å, particularly within key catalytic regions of LaSpdSyn. Energy components (electrostatic, van der Waals, and total potential and kinetic energies) were extracted from the NAMD logs. Additionally, all simulations were performed in triplicate (*n* = 3).

Statistical analyses were carried out in GraphPad Prism v.9 (GraphPad Software, California, USA). The Shapiro–Wilk test was used to determine dataset normality, and Gaussian curve fits were validated by residual distribution and R^2^ values. When comparing ACS47, ACS48, and ACS51 across species or simulation conditions, one-way ANOVA followed by Dunn’s post hoc test was used to determine statistically meaningful shifts in structural metrics or energetic trends.

#### 4.11.4. Binding Free Energy (ΔGbind) Assessments of Coumarins Against LaSpdSyn

Binding free energies were estimated using the CaFE plugin implemented in VMD, a workflow natively designed for trajectories generated with NAMD and CHARMM-based force fields [75]. Topology (.psf) and trajectories (.ddc) files were imported into VMD, where the protein–ligand complex, the isolated receptor, and the isolated ligand were defined through atom-selection masks. Hence, the CaFE was employed to perform MM/PBSA and, when indicated, LIE analyses across user-selected frames sampled from the production trajectories [76,77]. For each system, Δ*G_bind_* values were obtained by averaging the per-frame energy components (e.g., molecular mechanics terms, Poisson–Boltzmann solvation, and non-polar contributions), following the standard MM/PBSA pipeline.

#### 4.11.5. Assessment of SpdSyn Conservancy on the Leishmania Genus

To investigate the evolutionary and structural conservation of SpdSyn amongst representatives of the *Leishmania* genus, a multiple sequence alignment (MSA) was performed using the MUSCLE algorithm implemented in MEGA version 11.0 [78,79]. Six homologous sequences to LaSpdSyn were selected from a BLASTp search against the NCBI non-redundant (NR) protein database [80]. Homologs were chosen based on sequence identity, query coverage, and functional annotation as putative SpdSyn [53,81]. MUSCLE alignment was carried out with default gap open and extend parameters and with UPGMA clustering. Also, structural alignment was conducted using the MatchMaker tool within UCSF Chimera version 1.18 for comparative structural analysis [82]. The global alignment was based on the Needleman–Wunsch algorithm using the BLOSUM-62 scoring matrix [83]. A gap extension penalty of 1.0 was applied to balance alignment stringency with flexibility, and the algorithm was iteratively refined by pruning atom pairs exceeding 2.0 Å in spatial distance. Thus, RMSD was calculated between the pairwise aligned structures [84].

## 5. Conclusions

This study provides an evaluation of 7-prenyloxycoumarins as antileishmanial agents, combining in vitro screening, mammalian in vivo validation, and in silico mechanistic analysis. Among the tested compounds, the altissimacoumarin D analogues ACS47 and ACS48 were identified as the most promising leads. Both compounds significantly reduced parasite burden and lesion size in the hamster model at low doses (2.5 mg/kg), without inducing nephrotoxicity or hepatotoxicity. Importantly, this work advances the field by linking structural features of 7-prenyloxycoumarins to the polyamine biosynthetic pathway, with computational analyses consistently supporting spermidine synthase (SpdSyn) as a plausible molecular target conserved across *Leishmania* spp. From a translational perspective, 7-prenyloxycoumarins represent chemically tractable scaffolds for rational optimization toward oral or topical antileishmanial therapies. Future studies will focus on experimental validation of parasite and human SpdSyn inhibition, assessment of off-target interactions, and pharmacokinetic profiling to support preclinical development.

## Data Availability

The original contributions presented in this study are included in the article/Supplementary Materials. Further inquiries can be directed to the corresponding authors.

## Supplementary Materials

The following supporting information can be downloaded at: https://www.mdpi.com/article/10.3390/ph19030426/s1. Figure S1: Structural quality and surface properties of LaSpdSyn. Figure S2: Binding-site persistence and contact reorganization of ACS47, ACS48 and ACS51 within the catalytic cavity of LaSpdSyn along 500 ns MD simulations. Table S1: ADME properties of ACS47, ACS48, and ACS51. Table S2: Docking results of the most promising target candidates in *L. amazonensis* strain PH8.; Figure S3: Structural and sequence comparison of *Leishmania* spermidine synthase (LaSpdSyn) and the human homolog (HsSpdSyn), highlighting steric incompatibility with coumarins (ACS47/ACS48/ACS51) [44,45,62,63,82,85,86,87,88,89,90,91,92,93,94,95,96].
