# Supplementary material for: 7-Prenyloxycoumarins as Promising Antileishmanial Agents: In Vitro, In Vivo, and In Silico Evaluation Against Leishmania amazonensis
Source: Pharmaceuticals (Basel). 2026 Mar 5;19(3):426. doi: 10.3390/ph19030426 (PMC13029137; doi:10.3390/ph19030426)
Supplement: Supplementary file 1 [file pharmaceuticals-19-00426-s001.zip › pharmaceuticals-4151406-supplementary.pdf]

Supplementary material

Supplementary methods

***L. amazonensis* strain P88 and *Leishmania* spp. druggable target retrieval from structural databases and a molecular modelling pipeline**

Briefly, all L. amazonensis strain PH8 protein targets identified in the preliminary filtering stage (i.e., enzymes with essentiality evidence in survival pathways and other key aspects to parasite cell biology) were previously retrieved for structural databases PDB, Uniprot, and AlphaFold, comprising experimentally determined structures and predictions based on homology modelling. Additionally, model quality was assessed through complementary parameters retrieved from SWISS-MODEL or AlphaFold, including: (i) sequence identity relative to the template; (ii) the global model quality estimation (GMQE); (iii) the QMEAN and QMEANDisCo global scores [86]; (iv) and pLDDT [87]. Stereochemical validation included Ramachandran plot inspection using PROCHECK, emphasizing the proportion of residues in favored and allowed regions [87]. Additional structural evaluation was performed using MolProbity [89], providing the MolProbity score, clashscore, rotamer outliers, Cβ deviations, and detailed Ramachandran statistics.

Electrostatic surface potential calculations were carried out using APBS [89]. The modeled structure was pre-processed by PDB2PQR using the AMBER force field and default protonation parameters [90,91] to assign atomic charges and radii before electrostatic mapping. Hydrophobic surface distribution was examined according to the transmembrane-tendency scale of Zhao and London [92]. Solvent accessibility was estimated through two approaches: (i) residue-level relative solvent accessibility (RSA) extracted in UCSF Chimera using the "Relative Exposure of Amino Acids" protocol [82], and (ii) solvent exposure predictions obtained from NetSurfP-3.0 [93], using a 25% threshold to discriminate buried from exposed positions. Conserved functional domains were confirmed through Pfam, considering hits with E-value <1e−5 [94]. Ligand structures for ACS47, ACS48, and ACS51 were built and energy-minimized prior to docking. Three-dimensional visualization and structural inspections of the L. amazonensis strain PH8 target models and ligand-bound complexes were performed using UCSF Chimera (v1.18), PyMOL (v3.1; Schrödinger LLC) and BIOVIA Discovery Studio v. 2024 [52,53,83].

Ligand structures for ACS47, ACS48, and ACS51 were built and energy-minimized prior to docking. Three-dimensional visualization and structural inspections of the L. amazonensis strain PH8 target models and ligand-bound complexes were performed using UCSF Chimera (v1.18), PyMOL (v3.1; Schrödinger LLC) and BIOVIA Discovery Studio v. 2024 [51,52,82].

Target screening of *L. amazonensis* strain PH8 druggable targets by molecular docking

All L. amazonensis PH8 protein targets identified in the preliminary filtering stage (i.e., enzymes with essentiality evidence in polyamine metabolism and related survival pathways) were subjected to a standardized virtual screening workflow optimized to minimize ligand-size bias and pose degeneracy, as previously retrieved for structural databases PDB, Uniprot, and AlphaFold [44,45,94,95]. Each receptor was prepared at pH 7.4, with full protonation of catalytic residues, removal of crystallographic artifacts, and assignment of CHARMM-compatible parameters, prior to conversion to .pdbqt. Ligands ACS47, ACS48, and ACS51 were geometry-optimized and assigned Gasteiger charges, and a uniform search space was centered on the canonical active pocket of each enzyme, maintaining exhaustiveness, seed randomization, and local-search parameters constant across all runs to avoid search-space-driven variance for AutoDock Vina 1.2.0 [46,47]

An integrated decision rule evaluated docking poses: (i) primary hits required binding scores ≤ −8.0 kcal·mol^{-1}, mean RMSD (lower-bound clustering) ≤ 2.5 Å with SD ≤ 1.5 Å to ensure a compact, unimodal cluster, calculated dissociation constants (Kd) < 200 nM derived from ΔG, and per-atom scores ≤ −0.18 kcal·mol^{-1}·atom^{-1} to penalize large ligands that artificially inflate ΔG; (ii) secondary hits were accepted when binding scores fell between −7.2 and −7.99 kcal·mol^{-1}, RMSD l.b. ≤ 3.0 Å (SD ≤ 2.0 Å), Kd between 200–800 nM, and per-atom contributions ≤ −0.16; (iii) any docking result exhibiting multimodal or unstable pose distributions, operationally defined as RMSD l.b. mean > 4–5 Å or SD > 3 Å, was automatically discarded and re-docked to rule out artefacts from local-minima trapping [62,63,96].

Also, an additional exclusion criterion included favorable ΔG but per-atom scores > −0.15, which indicate energetically inflated but sterically unproductive solutions, and any scenario in which ACS47, ACS48, and ACS51 produced contradictory binding modes for the same receptor, flagging pocket instability or mis-assigned active sites. All numerical outputs (ΔG, Kd, pose clustering statistics, per-atom energies) were retained for quality control and cross-target comparability, ensuring that only chemically coherent, statistically compact, and mechanistically plausible coumarin–enzyme complexes proceeded to the downstream MD refinement stage.

Supplementary results

Target screening of *L. amazonensis* strain PH8 druggable targets by molecular docking

Across the target-screening pipeline, the combination of ΔG-derived affinities, cluster compactness and per-atom–normalized contributions consistently narrowed the initial receptor panel to a small set of druggable enzymes and membrane proteins, with cytochrome c peroxidase (Q4Q3K2) emerging as the dominant hit: all three coumarins met the primary thresholds (ΔG ≤ –8.0 kcal·mol^{-1}, Kd < 200 nM, RMSD ≤ 2.5 Å) and formed unusually tight pose families, with ACS48

reaching −8.82 kcal·mol^{-1} (Kd ≈ 21 nM; 2.69 ± 0.39 Å), ACS47 maintaining −8.56 kcal·mol^{-1} (50 nM; 3.65 ± 1.77 Å), and ACS51 retaining −8.44 kcal·mol^{-1} (66 nM; 1.47 ± 0.49 Å), yielding the best triad-level convergence and the cleanest per-atom performance (−0.276 to −0.162).

Aquaglyceroporin AQP1 (Q4Q6R2) also displayed a strong and reproducible signature, particularly for ACS48 (−8.70 kcal·mol^{-1}; 45 nM; 2.29 ± 0.25 Å) and ACS51 (−8.00 kcal·mol^{-1}; 138 nM; 1.63 ± 0.45 Å), while ACS47 remained within a secondary cut (−7.58 kcal·mol^{-1}; 279 nM; 1.48 ± 0.38 Å); the narrow dispersions (<0.5–0.6 Å for two ligands) and stable per-atom ratios supported their progression to membrane-embedded MD in PMm (asymmetric mammalian plasma membrane-like composition) biomembrane (e.g., POPC, POPE, POPS, and POPG derivatives and CHOL) to assess pore-facing interactions under microhydrated constraints (Wu et al., 2014; Lee et al., 2016).

In the polyamine-biosynthesis axis, spermidine synthase (A0A0B4ULH7; LaSpdSyn) and AdoMetDC (Q25264) showed internally consistent but slightly weaker profiles: ACS48 repeatedly yielded the highest quality clusters in both enzymes (−7.92 kcal·mol^{-1}, 1.41 ± 0.70 Å, 154 nM in SpdS; −7.76 kcal·mol^{-1}, 1.83 ± 0.56 Å, 237 nM in AdoMetDC), while ACS51 maintained comparable ΔG in SpdS (−7.92 kcal·mol^{-1}; 154 nM; 2.54 ± 1.06 Å) and acceptable dispersion in AdoMetDC (−7.26 kcal·mol^{-1}; 428 nM; 2.12 ± 0.63 Å). Although ACS47 ranked last within the polyamine enzymes (SpdS: −7.32 kcal·mol^{-1}, 412 nM, 2.86 ± 1.56 Å; AdoMetDC: −6.98 kcal·mol^{-1}, 806 nM, 2.64 ± 1.01 Å), the preservation of coherent binding modes justified its inclusion for comparative MD given the essentiality and metabolic chokepoint nature of this pathway.

Cytochrome bc1 (P14548) displayed uniformly compact clusters across the three ligands (RMSD 1.80–1.93 Å) but only moderate affinities (−7.64 to −6.96 kcal·mol^{-1}; 256–774 nM), positioning it as a secondary mitochondrial-disruption candidate. Taken together, the screening stage converged on four high-value systems for MD validation: cytochrome c peroxidase, AQP1, spermidine synthase, and AdoMetDC, selected not solely by ΔG but by the joint reproducibility of pose topology, RMSD variance, sub-200-nM Kd thresholds, and the presence of pathway-relevant or structurally tractable pockets suitable for downstream mechanistic interrogation.

LaSpdSyn quality, surface, and biochemical properties

LaSpdSyn showed the expected structural and stereochemical quality for a conserved polyamine-pathway enzyme of the Leishmania genus, with 95–98% of residues occupying favored and allowed regions of the Ramachandran plot (Supplementary figure 1A), and no outliers detected within the catalytic pocket, which remained free of steric clashes and did not require rotameric adjustments upon docking of ACS47, ACS48 or ACS51. AlphaFold-derived pLDDT scores remained high across the six α-helices (α1–α6) and two β-strands (β1–β2), with values frequently >85 for core secondary-structure elements (Supplementary figure 1B),

matching the well-packed hydrophobic core revealed by solvent accessibility and hydrophobicity maps (Supplementary figure 1C–D).

In addition, the pLDDT–RSA overlay indicated that exposed residues across loops (e.g., α2–β1, β2–α4, α5–α6) naturally showed lower confidence or higher RSA, whereas regions shaping the catalytic cleft, such as α1, β1, α3, and α4, remained structurally rigid (Supplementary figure 1 E). Also, ProtPi calculations confirmed a compact 110-aa protein (11.55 kDa) with acidic character (pI = 5.23; net charge −3.62 at pH 7.4) and a composition dominated by hydrophobic residues (≈50%, with Val = 13.64%, Gly = 12.73% and Ala = 8.18%), consistent with the enzyme's recessed binding pocket and the coumarin-friendly apolar interior.

Compounds' pose depiction at the half and the end of MD simulations

Across the 500 ns simulations, all three compounds remained persistently anchored within the catalytic cavity of LaSpdSyn, sustaining a conserved interaction network centered on the β1 segment (V26–D30) and the α4/α4–α5 region (F65, V66, R68, V73–I77), which together form the hydrophobic–acidic microenvironment that accommodates the coumarin scaffold (Supplementary figure 2). ACS47 showed the most rigid and time-invariant binding mode, preserving virtually identical contacts at 250 and 500 ns, including the consistent engagement of V26, L28 and V29 (β1) together with the α4 cluster (F65, V66, R68) and loop α4–β3 residues (V73, Y74, V76, I77), in agreement with its lowest ligand-RMSD (1.52 ± 0.26 Å; ****$p < 0.0001$) and minimal backbone drift relative to ACS48/ACS51 (Supplementary figure 2A-B).

Additionally, ACS48 reproduced the same anchoring elements but adopted a slightly more open pose at 500 ns, with its prenyl chain projecting toward the C-terminal portion of the cavity while the coumarin core remained locked toward the α1–β1 interface, consistent with its higher ligand mobility (1.65 ± 0.29 Å) and backbone RMSD (2.01 ± 0.37 Å) (Supplementary figure 2C-D). Also, ACS51, bearing the longest aliphatic chain, engaged the broadest set of residues, extending beyond β1 and α4 to distal hydrophobic pockets at 500 ns, reflecting its increased internal motion (1.72 ± 0.28 Å) while still preserving the same β1/α4 anchoring observed for ACS47 and ACS48 (Supplementary figure 2E-F). Across all complexes, neither displacement of the coumarin nucleus nor loss of the β1–α4 recognition motif was detected between 250 and 500 ns, corroborating the pose-stability metrics and supporting LaSpdSyn as a viable molecular target whose active-site topology accommodates the three active compounds through a structurally conserved and time-persistent mechanism of interaction.

Moreover, Supplementary Figure 3 summarizes the sequence–structure comparison between the *Leishmania* putative spermidine synthase (LaSpdSyn; annotation-based locus) and the human homolog (HsSpdSyn; PDB 2O05), integrating a full-length alignment (310 aa),

identity/similarity metrics, and active-site geometry. Across the *Leishmania* spp. sequences examined, LaSpdSyn shares low identity with HsSpdSyn (14.84–16.45%; 46–51 identical residues/310 aa) and uniformly low similarity (21.29%; 15–20 similar residues/310 aa), consistent with substantial divergence at the primary-sequence level despite conservation of the aminopropyltransferase fold seen upon structural superposition. Notably, pocket-focused overlays indicate that the altissimacoumarins (ACS47/ACS48/ACS51) are sterically incompatible with HsSpdSyn, with the dominant clashes mapping to residues L98 and I99, supporting the view that local “gatekeeper” features in the human binding pocket can limit accommodation of these ligands even under an overall conserved fold framework.

Supplementary figures and tables with captions

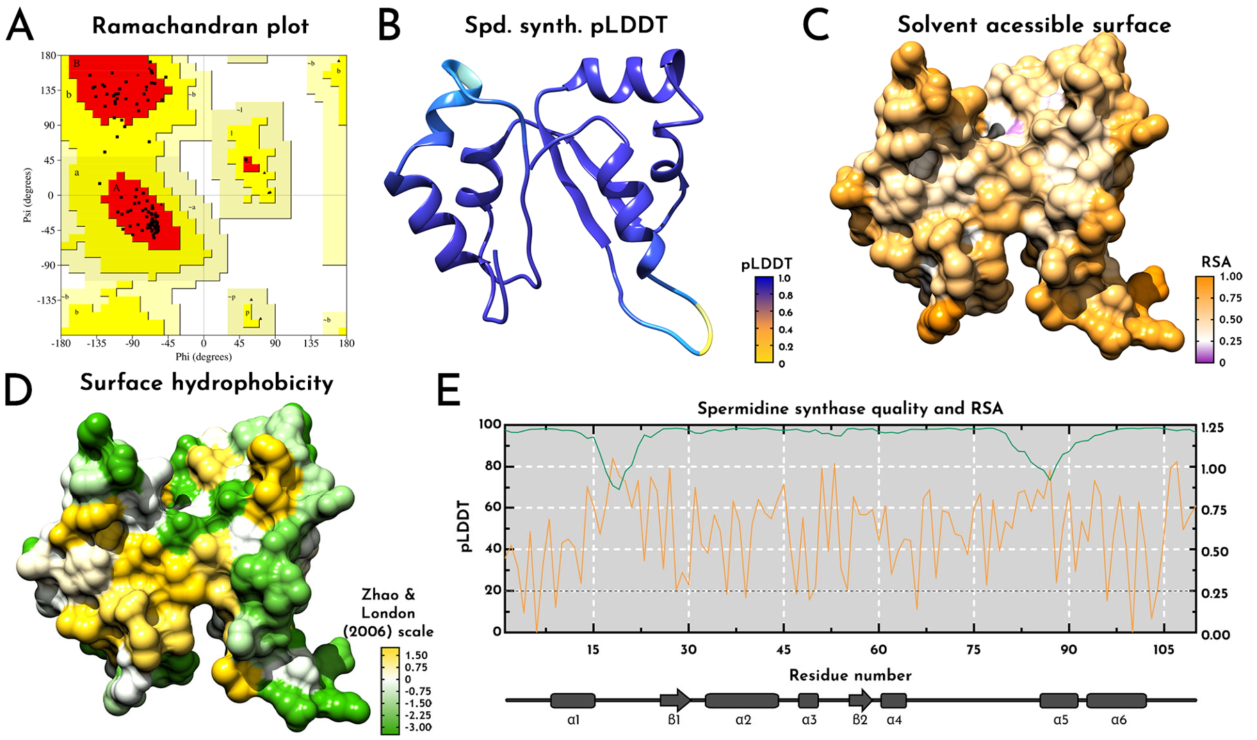

Figure S1. Structural quality and surface properties of LaSpdSyn. **A.** Ramachandran plot. PROCHECK analysis places 89.8% of residues in the most-favored regions and the remaining 10.2% in additionally allowed zones, with no residues in generously allowed or disallowed areas. Side-chain geometry remained within expected limits (five favorable outliers; zero “worse”), and no bad contacts were detected. G-factors for dihedrals (–0.02) and covalent geometry (0.31) indicate a well-behaved model. **B.** pLDDT confidence. The AlphaFold model shows uniformly high confidence across the fold (pLDDT = 94.82 ± 6.41). Only short terminal segments fall below 90. **C.** Solvent-accessible surface (RSA). Residue-wise RSA values span from fully buried to fully exposed (mean RSA = 0.605 ± 0.252), with the catalytic pocket forming a predominantly low-RSA, recessed cavity. **D.** Surface hydrophobicity. Mapping onto the Zhao–London (2006) scale reveals an extended hydrophobic belt traversing α2–α4 and adjoining loops, consistent with the expected polyamine-binding groove, while distal surfaces exhibit mixed or polar character. **E.** pLDDT–RSA profile across the sequence. Per-residue traces show that well-packed secondary-structure elements (α1–α6, β1–β2) combine high confidence (pLDDT > 90) and low accessibility (RSA < 0.4), whereas exposed coil regions display higher RSA dispersion (0.00–1.05) and larger pLDDT fluctuations. The overall coefficient of variation highlights the compactness of the model (pLDDT CV = 6.76%) versus the broader dynamic range of solvent exposure (RSA CV = 41.6%).

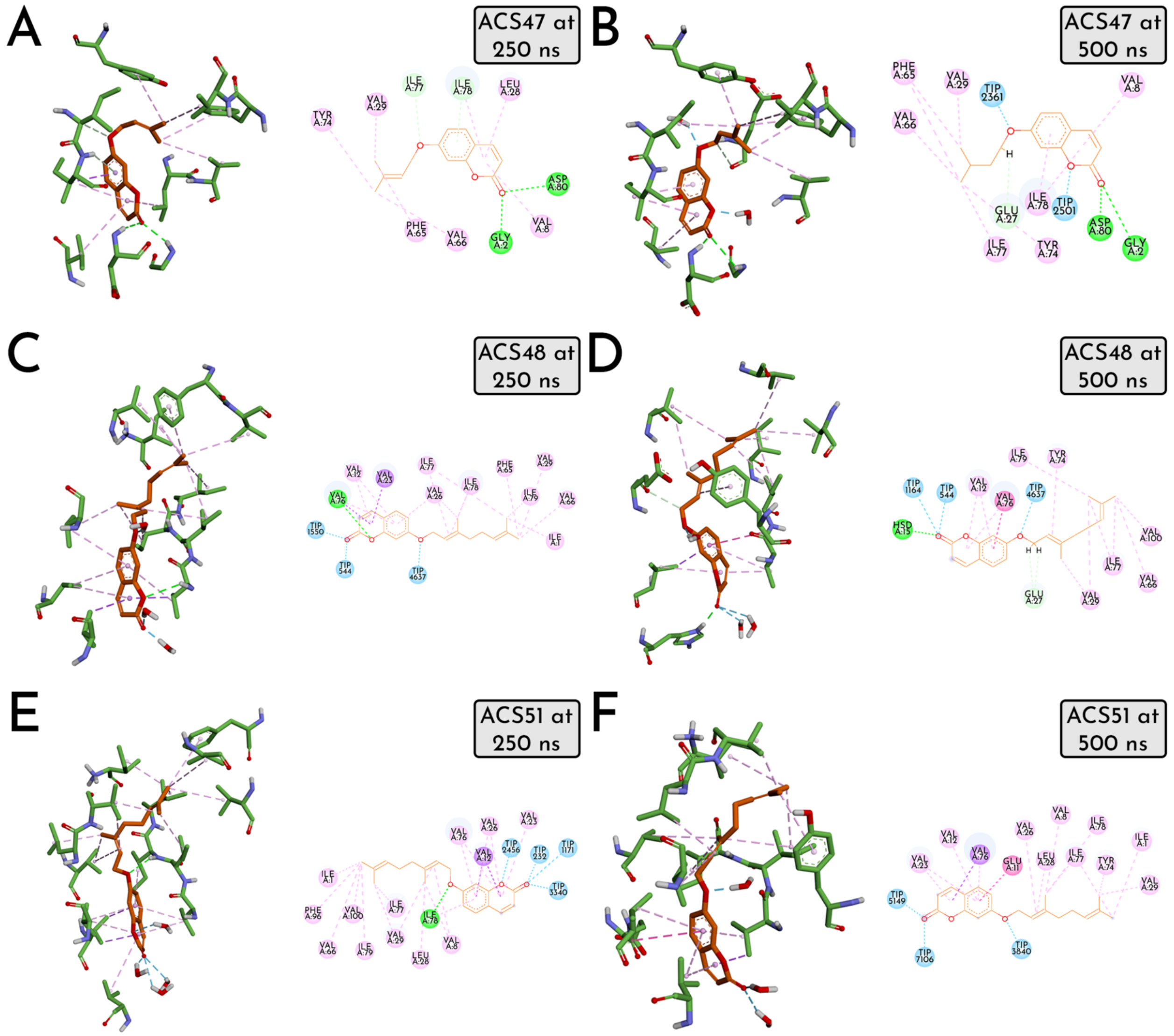

Figure S2. Binding-site persistence and contact reorganization of ACS47, ACS48 and ACS51 within the catalytic cavity of LaSpdSyn along 500 ns MD simulations. **A–B.** ACS47 at 250 ns and 500 ns. ACS47 maintains a stable pose across time, preserving contacts with residues from α1/β1 and α4–α5 regions, notably V26 (β1), L28 (β1), V29 (β1), D30 (β1) and hydrophobic side-chain packing involving F65 (α4), V66 (α4) and R68 (α4). Over the 250→500-ns interval, the coumarin core remains anchored within the same sub-pocket, and the prenyl tail retains proximity to loop α4–α5 residues (e.g., V73, Y74, V76, I77). No major loss of contacts is evident, consistent with the lowest ligand-RMSD variability observed for ACS47 (mean 1.52 ± 0.26 Å; ****$p < 0.0001$ vs. ACS48/ACS51). **C–D.** ACS48 at 250 ns and 500 ns. ACS48 sustains a similar anchoring pattern to ACS47, with recurrent interactions at β1 (V26, L28, V29), α4 (F65, V66, R68) and loop α4–α5 (V73, Y74, V76). At 500 ns, the ligand shows a more open conformation, with the aliphatic chain extending toward the C-terminal region, while the coumarin nucleus remains oriented toward the α1/β1 face. This subtle repositioning matches the higher ligand RMSD (1.65 ± 0.29 Å) and the protein-backbone drift previously quantified for ACS48 (2.01 ± 0.37 Å; ANOVA, ****$p < 0.0001$). **E–F.** ACS51 at 250 ns and 500 ns. ACS51 displays the broadest interaction footprint among the three compounds, repeatedly involving β1 (V26, L28, V29, D30) and hydrophobic nodes in α4 (F65, V66, R68) and loop α4–α5 (V73, Y74, V76, I77). Its extended geranyl chain engages more distal hydrophobic residues at 500 ns, compatible with its slightly higher internal mobility (ligand RMSD 1.72 ±

0.28 Å) and backbone deviation (2.01 ± 0.34 Å). The conserved coumarin–β1/α4 interactions observed at both 250 and 500 ns indicate a persistent anchoring mode shared with ACS47 and ACS48.

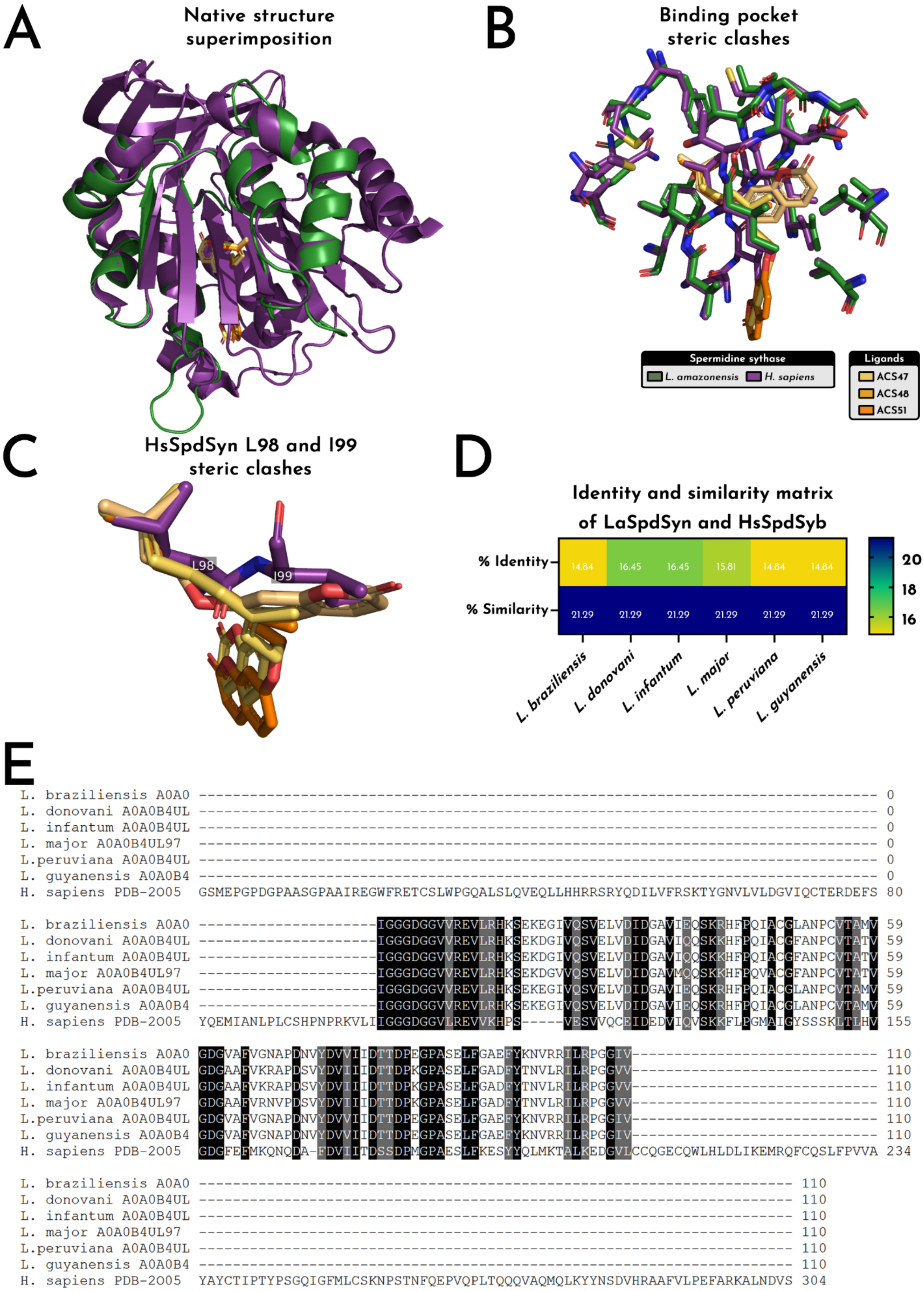
A
Native structure superimposition
B
Binding pocket steric clashes
Spermidine sythase
L. amazonensis
H. sapiens
Ligands
ACS47
ACS48
ACS51
C
HsSpdSyn L98 and I99 steric clashes
L98
I99
D
Identity and similarity matrix of LaSpdSyn and HsSpdSyb
% Identity
% Similarity
14.84
16.45
16.45
15.81
14.84
14.84
21.29
21.29
21.29
21.29
21.29
21.29
20
18
16
L. braziliensis
L. donovani
L. infantum
L. major
L. peruviana
L. guyanensis
E
L. braziliensis A0A0 -- 0
L. donovani A0A0B4UL -- 0
L. infantum A0A0B4UL -- 0
L. major A0A0B4UL97 -- 0
L.peruviana A0A0B4UL -- 0
L. guyanensis A0A0B4 -- 0
H. sapiens PDB-2005 GSMEPGPDGPAASGPAAIREGWFRETCSLWPGQALSLQVEQLLHHRRSRYQDILVFRSKTYGNVLVLDGVIQCTERDEFS 80
L. braziliensis A0A0 ---------------------IGGGDGGVVREVLRHKSEKEGIVQSVELVDIDGAVIEQSKRHFPQIACGLANPCVTAMV 59
L. donovani A0A0B4UL ---------------------IGGGDGGVVREVLRHKSEKDGIVQSVELVDIDGAVIQQSKKHFPQIACGFANPCVTATV 59
L. infantum A0A0B4UL ---------------------IGGGDGGVVREVLRHKSEKDGIVQSVELVDIDGAVIQQSKKHFPQIACGFANPCVTATV 59
L. major A0A0B4UL97 ---------------------IGGGDGGVVREVLRHKSEKDGVVQSVELVDIDGAVMQQSKKHFPQVACGFANPCVTATV 59
L.peruviana A0A0B4UL ---------------------IGGGDGGVVREVLRHKSEKEGIVQSVELVDIDGAVIEQSKRHFPQIACGLANPCVTAMV 59
L. guyanensis A0A0B4 ---------------------IGGGDGGVVREVLRHKSEKEGIVQSVELVDIDGAVIEQSKRHFPQIACGLANPCVTAMV 59
H. sapiens PDB-2005 YQEMIANLPLCSHPNPRKVLIIGGGDGGVLREVVKHPS-----VESVVQCEIDEDVIQVSKKFLPGMAIGYSSSKLTLHV 155
L. braziliensis A0A0 GDGVAFVGNAPDNVYDVVIIDTTDPEGPASELFGAEFYKNVRRILRPGGIV----------------------------- 110
L. donovani A0A0B4UL GDGAAFVKRAPDSVYDVIIIDTTDPKGPASELFGADFYTNVLRILRPGGVV----------------------------- 110
L. infantum A0A0B4UL GDGAAFVKRAPDSVYDVIIIDTTDPKGPASELFGADFYTNVLRILRPGGVV----------------------------- 110
L. major A0A0B4UL97 GDGAAFVRNVPDSVYDVIIIDTTDPKGPASELFGADFYTNVLRILRPGGVV----------------------------- 110
L.peruviana A0A0B4UL GDGVAFVGNAPDNVYDVVIIDTTDPEGPASELFGAEFYKNVRRILRPGGIV----------------------------- 110
L. guyanensis A0A0B4 GDGVAFVGNAPDNVYDVVIIDTTDPEGPASELFGAEFYKNVRRILRPGGIV----------------------------- 110
H. sapiens PDB-2005 GDGFEFMKQNQDA-FDVIITDSSDPMGPAESLFKESYYQLMKTALKEDGVLCCQGECQWLHLDLIKEMRQFCQSLFPVVA 234
L. braziliensis A0A0 -- 110
L. donovani A0A0B4UL -- 110
L. infantum A0A0B4UL -- 110
L. major A0A0B4UL97 -- 110
L.peruviana A0A0B4UL -- 110
L. guyanensis A0A0B4 -- 110
H. sapiens PDB-2005 YAYCTIPTYPSGQIGFMLCSKNPSTNFQEPVQPLTQQQVAQMQLKYYNSDVHRAAFVLPEFARKALNDVS 304

Figure S3. Structural and sequence comparison of *Leishmania* spermidine synthase (LaSpdSyn) and the human homolog (HsSpdSyn), highlighting steric incompatibility with coumarins (ACS47/ACS48/ACS51). **A.** Native structure superimposition. LaSpdSyn (green) and HsSpdSyn (purple) show an overall conserved aminopropyltransferase fold, supporting cross-species homology despite local deviations around the active-site region. **B.** Binding pocket steric clashes. When ACS47 (yellow), ACS48 (gold), and ACS51 (orange) are positioned in the binding site, the human pocket presents pronounced steric overlaps relative to the parasite model, consistent with reduced geometric accommodation in HsSpdSyn. **C.** HsSpdSyn L98 and I99 steric clashes. The major clashes map to HsSpdSyn residues L98 and I99, which intrude into the ligand-occupied volume for all three ACS ligands, indicating a putative "gatekeeper" constraint in the human enzyme. **D.** Identity and similarity matrix of LaSpdSyn and HsSpdSyn. Pairwise comparison shows low identity across *Leishmania* spp. vs human (14.84–16.45%) and uniformly low similarity (21.29%), consistent with substantial divergence at the sequence level. **E.** Sequence alignment. The alignment highlights limited conservation overall and supports the identity/similarity metrics, with short conserved motifs embedded within broader non-conserved regions, in line with the structural/steric differences observed near the binding pocket.

Table S1. ADME properties of biologically active coumarins ACS47, ACS48, and ACS51.

Parameter	ACS47	ACS48	ACS51
Molecular formula	$C_{14}H_{14}O_5$	$C_{19}H_{22}O_3$	$C_{21}H_{26}O_5$
Molecular weight ($g \cdot mol^{-1}$)	230.26	298.38	358.43
Heavy atoms	17	22	26
Aromatic heavy atoms	10	10	10
Fraction Csp^3	0.21	0.32	0.38
Rotatable bonds	3	6	8
H-bond acceptors	3	3	5
H-bond donors	0	0	0
Topological polar surface area (TPSA, $Å^2$)	39.44	39.44	57.90
Molar refractivity	67.73	91.29	104.28
Log P (consensus)	03.03	4.51	4.42
Water solubility (Log S, ESOL)	−3.55 (soluble)	−4.84 (moderately soluble)	−5.08 (moderately soluble)
GI absorption	High	High	High
BBB permeant	Yes	Yes	Yes
P-gp substrate	No	No	No

CYP1A2 inhibitor	Yes	Yes	No
CYP2C19 inhibitor	Yes	Yes	No
CYP2C9 inhibitor	No	No	No
CYP2D6 inhibitor	No	No	No
CYP3A4 inhibitor	No	No	No
Log Kp (skin permeation, cm/s)	−5.40	−4.50	−4.81
Lipinski compliance	Yes (0 violation)	Yes (0 violation)	Yes (0 violation)
Ghose, Veber, Egan, and Muegge rules	All Yes	All Yes (except Muegge: 1 viol.)	All Yes (except Muegge: 1 viol.)
Bioavailability score	0.55	0.55	0.55
PAINS alerts	0	0	0
Brenk alerts	2 (coumarin, isolated alkene)	2 (coumarin, isolated alkene)	2 (coumarin, isolated alkene)
Lead-likeness	1 viol. (MW < 250)	1 viol. (XLOGP3 > 3.5)	3 viol. (MW > 350, Rotors > 7, XLOGP3 > 3.5)
Synthetic accessibility	2.92	3.43	3.98

Table S2. Docking results of the most promising target candidates in *L. amazonensis* strain PH8

Receptor	Pathway	Ligand	Binding score (kcal/mol)	RMSD (mean ± SD, Å)	Kd (nM)	Normalized score (kcal/mol per atom)
A0A0B4ULH7_Spermidine synthase	Polyamine biosynthesis	ACS47 (31 atoms)	−7.32 ± 0.48	2.86 ± 1.56	4.12×10^2 nM	−0.236 kcal/mol · atom^{-1}
A0A0B4ULH7_Spermidine synthase	Polyamine biosynthesis	ACS48 (44 atoms)	−7.92 ± 0.25	1.41 ± 0.70	1.54×10^2 nM	−0.180 kcal/mol · atom^{-1}
A0A0B4ULH7_Spermidine synthase	Polyamine biosynthesis	ACS51 (52 atoms)	−7.92 ± 0.42	2.54 ± 1.06	1.54×10^2 nM	−0.152 kcal/mol · atom^{-1}
A0A6L0XET1_Succinate dehydrogenase	Tricarboxylic acid (TCA) cycle / Electron transport chain	ACS47 (31 atoms)	−7.9 ± 0.29	1.53 ± 0.80	1.59×10^2 nM	−0.255 kcal/mol·atom^{-1}
A0A6L0XET1_Succinate dehydrogenase	Tricarboxylic acid (TCA) cycle / Electron transport chain	ACS48 (44 atoms)	−8.26 ± 0.62	2.78 ± 1.01	8.93×10^1 nM	−0.188 kcal/mol·atom^{-1}

A0A6L0XET1_Succinate dehydrogenase	Tricarboxylic acid (TCA) cycle / Electron transport chain	ACS51 (52 atoms)	−7.16 ± 0.69	12.98 ± 9.29	5.76×10^3 nM	−0.138 kcal/mol·atom^{-1}
A4HCL7_tryparedoxin peroxidase	Antioxidant defense / Trypanothione-dependent redox metabolism	ACS47 (31 atoms)	−4.99 ± 0.32	6.71 ± 6.33	2.61×10^4 nM	−0.161 kcal/mol·atom^{-1}
A4HCL7_tryparedoxin peroxidase	Antioxidant defense / Trypanothione-dependent redox metabolism	ACS48 (44 atoms)	−5.90 ± 0.18	7.87 ± 5.87	7.72×10^3 nM	−0.134 kcal/mol·atom^{-1}
A4HCL7_tryparedoxin peroxidase	Antioxidant defense / Trypanothione-dependent redox metabolism	ACS51 (52 atoms)	−5.12 ± 0.19	12.69 ± 9.37	1.45×10^4 nM	−0.098 kcal/mol·atom^{-1}
A4HPV5_Alternative oxidase-AOX	Mitochondrial respiratory chain (alternative electron transport)	ACS47 (31 atoms)	−7.06 ± 0.45	1.76 ± 0.76	6.84×10^2 nM	−0.228 kcal/mol·atom^{-1}
A4HPV5_Alternative oxidase-AOX	Mitochondrial respiratory chain (alternative electron transport)	ACS48 (44 atoms)	−7.98 ± 0.35	5.28 ± 7.38	1.46×10^2 nM	−0.181 kcal/mol·atom^{-1}
A4HPV5_Alternative oxidase-AOX	Mitochondrial respiratory chain (alternative electron transport)	ACS51 (52 atoms)	−7.60 ± 0.29	2.77 ± 1.31	2.78×10^2 nM	−0.146 kcal/mol·atom^{-1}
A4HYH2_Cysteine peptidase A	Proteolysis / Lysosomal degradation	ACS47 (31 atoms)	−5.44 ± 0.20	10.97 ± 10.47	9.82×10^3 nM	−0.175 kcal·mol^{-1}·atom^{-1}
A4HYH2_Cysteine peptidase A	Proteolysis / Lysosomal degradation	ACS48 (44 atoms)	−5.68 ± 0.13	3.12 ± 2.98	7.22×10^3 nM	−0.129 kcal·mol^{-1}·atom^{-1}
A4HYH2_Cysteine peptidase A	Proteolysis / Lysosomal degradation	ACS51 (52 atoms)	−5.60 ± 0.11	2.94 ± 1.26	7.79×10^3 nM	−0.108 kcal·mol^{-1}·atom^{-1}

A4I6S0_Pentamidine resistance protein 1	Drug efflux / Polyamine transport system	ACS47 (31 atoms)	−6.22 ± 0.33	4.20 ± 2.25	2.53 × 10³ nM	−0.201 kcal/mol·atom⁻¹
A4I6S0_Pentamidine resistance protein 1	Drug efflux / Polyamine transport system	ACS48 (44 atoms)	−5.64 ± 0.35	22.44 ± 9.13	7.10 × 10³ nM	−0.128 kcal/mol·atom⁻¹
A4I6S0_Pentamidine resistance protein 1	Drug efflux / Polyamine transport system	ACS51 (52 atoms)	−6.58 ± 0.33	3.76 ± 1.99	1.30 × 10³ nM	−0.127 kcal/mol·atom⁻¹
A5JV94_Trypanothione synthetase	Trypanothione biosynthesis / Redox metabolism	ACS47 (31 atoms)	−6.44 ± 0.33	5.00 ± 3.74	1.88 × 10³ nM	−0.208 kcal/mol·atom⁻¹
A5JV94_Trypanothione synthetase	Trypanothione biosynthesis / Redox metabolism	ACS48 (44 atoms)	−5.78 ± 0.13	23.23 ± 23.64	6.75 × 10³ nM	−0.131 kcal/mol·atom⁻¹
A5JV94_Trypanothione synthetase	Trypanothione biosynthesis / Redox metabolism	ACS51 (52 atoms)	−6.36 ± 0.13	22.47 ± 12.80	2.23 × 10³ nM	−0.122 kcal/mol·atom⁻¹
B5APK2_Deoxyhypusine synthase	eIF5A hypusination / polyamine-derived post-translational modification	ACS47 (31 atoms)	−7.12 ± 0.22	1.48 ± 0.92	5.98 × 10³	−0.230 kcal/mol · atom⁻¹
B5APK2_Deoxyhypusine synthase	eIF5A hypusination / polyamine-derived post-translational modification	ACS48 (44 atoms)	−7.34 ± 0.26	2.86 ± 1.70	4.12 × 10³	−0.167 kcal/mol · atom⁻¹
B5APK2_Deoxyhypusine synthase	eIF5A hypusination / polyamine-derived post-translational modification	ACS51 (52 atoms)	−6.98 ± 0.15	1.36 ± 0.91	7.58 × 10³	−0.134 kcal/mol · atom⁻¹
E9ADT5_Cysteine peptidase C	Proteolysis / Lysosomal degradation	ACS47 (31 atoms)	−5.60 ± 0.13	23.31 ± 1.00	7.90 × 10³ nM	−0.181 kcal·mol⁻¹·atom⁻¹
E9ADT5_Cysteine peptidase C	Proteolysis / Lysosomal degradation	ACS48 (44 atoms)	−5.16 ± 0.27	12.43 ± 13.84	2.00 × 10⁴ nM	−0.117 kcal·mol⁻¹·atom⁻¹

E9ADT5_Cysteine peptidase C	Proteolysis / Lysosomal degradation	ACS51 (52 atoms)	−5.20 ± 0.20	12.40 ± 13.04	1.78 × 10^4 nM	−0.100 kcal·mol^{-1}·$atom^{-1}$
E9AEY0_Metacaspase-MCA5	Programmed cell death / Protease-mediated apoptosis	ACS47 (31 atoms)	−6.48 ± 0.33	1.52 ± 0.69	1.75 × 10^3 nM	−0.209 kcal/mol·$atom^{-1}$
E9AEY0_Metacaspase-MCA5	Programmed cell death / Protease-mediated apoptosis	ACS48 (44 atoms)	−6.70 ± 0.09	1.48 ± 0.33	1.02 × 10^3 nM	−0.152 kcal/mol·$atom^{-1}$
E9AEY0_Metacaspase-MCA5	Programmed cell death / Protease-mediated apoptosis	ACS51 (52 atoms)	−6.12 ± 0.26	2.27 ± 0.60	3.37 × 10^3 nM	−0.118 kcal/mol·$atom^{-1}$
E9AFX2_IPCS-ceramide inositolphosphotransferase	Sphingolipid biosynthesis / Membrane lipid metabolism	ACS47 (31 atoms)	−6.04 ± 0.16	2.12 ± 0.85	3.83 × 10^3 nM	−0.195 kcal/mol·$atom^{-1}$
E9AFX2_IPCS-ceramide inositolphosphotransferase	Sphingolipid biosynthesis / Membrane lipid metabolism	ACS48 (44 atoms)	−6.98 ± 0.33	2.96 ± 1.83	7.58 × 10^2 nM	−0.159 kcal/mol·$atom^{-1}$
E9AFX2_IPCS-ceramide inositolphosphotransferase	Sphingolipid biosynthesis / Membrane lipid metabolism	ACS51 (52 atoms)	−6.44 ± 0.37	2.54 ± 0.38	1.88 × 10^3 nM	−0.124 kcal/mol·$atom^{-1}$
E9BRC0_DNA-topoisomerase I_2B9S	DNA replication / DNA supercoiling relaxation	ACS47 (31 atoms)	−5.88 ± 0.29	21.33 ± 3.87	4.86 × 10^3 nM	−0.190 kcal/mol·$atom^{-1}$
E9BRC0_DNA-topoisomerase I_2B9S	DNA replication / DNA supercoiling relaxation	ACS48 (44 atoms)	−6.46 ± 0.17	17.56 ± 1.02	1.61 × 10^3 nM	−0.147 kcal/mol·$atom^{-1}$
E9BRC0_DNA-topoisomerase I_2B9S	DNA replication / DNA supercoiling relaxation	ACS51 (52 atoms)	−5.94 ± 0.16	36.13 ± 2.95	4.12 × 10^3 nM	−0.114 kcal/mol·$atom^{-1}$
E9BRW1_fumarate reductase	Tricarboxylic acid cycle / Anaerobic respiration	ACS47 (31 atoms)	−6.90 ± 0.37	3.96 ± 4.22	8.48 × 10^2 nM	−0.223 kcal/mol·$atom^{-1}$

E9BRW1_fumarate reductase	Tricarboxylic acid cycle / Anaerobic respiration	ACS48 (44 atoms)	−6.76 ± 0.23	18.93 ± 13.86	1.24 × 10^3 nM	−0.154 kcal/mol·atom^{-1}
E9BRW1_fumarate reductase	Tricarboxylic acid cycle / Anaerobic respiration	ACS51 (52 atoms)	−6.38 ± 0.33	11.86 ± 11.38	2.64 × 10^3 nM	−0.123 kcal/mol·atom^{-1}
O15826_Putative deoxyuridine triphosphatase-dUTPase_2YAY	Pyrimidine metabolism / dUTP hydrolysis	ACS47 (31 atoms)	−6.56 ± 0.24	1.87 ± 0.86	1.62 × 10^3 nM	−0.212 kcal/mol·atom^{-1}
O15826_Putative deoxyuridine triphosphatase-dUTPase_2YAY	Pyrimidine metabolism / dUTP hydrolysis	ACS48 (44 atoms)	−7.02 ± 0.25	1.29 ± 0.57	7.15 × 10^2 nM	−0.160 kcal/mol·atom^{-1}
O15826_Putative deoxyuridine triphosphatase-dUTPase_2YAY	Pyrimidine metabolism / dUTP hydrolysis	ACS51 (52 atoms)	−6.42 ± 0.15	1.99 ± 0.56	2.36 × 10^3 nM	−0.123 kcal/mol·atom^{-1}
O76269_Nucleoside transporter 1-NT1	Purine/pyrimidine salvage pathway / Nucleoside uptake	ACS47 (31 atoms)	−6.66 ± 0.22	2.83 ± 0.54	1.41 × 10^3 nM	−0.215 kcal/mol·atom^{-1}
O76269_Nucleoside transporter 1-NT1	Purine/pyrimidine salvage pathway / Nucleoside uptake	ACS48 (44 atoms)	−6.92 ± 0.32	2.08 ± 0.11	9.12 × 10^2 nM	−0.157 kcal/mol·atom^{-1}
O76269_Nucleoside transporter 1-NT1	Purine/pyrimidine salvage pathway / Nucleoside uptake	ACS51 (52 atoms)	−6.88 ± 0.40	11.80 ± 10.24	9.73 × 10^2 nM	−0.132 kcal/mol·atom^{-1}
O96394_Arginase	Urea cycle and L-arginine metabolism	ACS47 (31 atoms)	−5.66 ± 0.23	9.85 ± 11.20	7.73 × 10^3 nM	−0.183 kcal/mol·atom^{-1}
O96394_Arginase	Urea cycle and L-arginine metabolism	ACS48 (44 atoms)	−5.68 ± 0.14	13.39 ± 11.20	7.42 × 10^3 nM	−0.129 kcal/mol·atom^{-1}
O96394_Arginase	Urea cycle and L-arginine metabolism	ACS51 (52 atoms)	−5.80 ± 0.17	2.92 ± 1.73	5.77 × 10^3 nM	−0.112 kcal/mol·atom^{-1}

O96439_Adenosine kinase_ADK	Purine metabolism / adenosine salvage & phosphorylation	ACS47 (31 atoms)	−6.60 ± 0.29	4.21 ± 4.30	1.44×10^4	−0.213 kcal/mol · atom^{-1}
O96439_Adenosine kinase_ADK	Purine metabolism / adenosine salvage & phosphorylation	ACS48 (44 atoms)	−7.22 ± 0.40	2.33 ± 1.32	5.05×10^3	−0.164 kcal/mol · atom^{-1}
O96439_Adenosine kinase_ADK	Purine metabolism / adenosine salvage & phosphorylation	ACS51 (52 atoms)	−6.76 ± 0.23	2.22 ± 1.26	1.10×10^4	−0.130 kcal/mol · atom^{-1}
P14548_Cytochrome bc1_complex IV	Electron transport chain / oxidative phosphorylation	ACS47 (31 atoms)	−6.96 ± 0.13	1.86 ± 0.88	7.74×10^2	−0.225 kcal/mol · atom^{-1}
P14548_Cytochrome bc1_complex IV	Electron transport chain / oxidative phosphorylation	ACS48 (44 atoms)	−7.64 ± 0.19	1.80 ± 0.81	2.56×10^2	−0.174 kcal/mol · atom^{-1}
P14548_Cytochrome bc1_complex IV	Electron transport chain / oxidative phosphorylation	ACS51 (52 atoms)	−7.18 ± 0.11	1.93 ± 0.43	4.91×10^2	−0.138 kcal/mol · atom^{-1}
P16126_DHFR	Folate biosynthesis	ACS47 (31 atoms)	−7.10 ± 0.22	1.50 ± 0.77	6.03×10^2 nM	−0.229 kcal/mol · atom^{-1}
P16126_DHFR	Folate biosynthesis	ACS48 (44 atoms)	−7.58 ± 0.15	1.68 ± 0.89	2.80×10^2 nM	−0.172 kcal/mol · atom^{-1}
P16126_DHFR	Folate biosynthesis	ACS51 (52 atoms)	−6.68 ± 0.38	2.71 ± 1.03	1.33×10^3 nM	−0.128 kcal/mol · atom^{-1}
P21441_PGPA-Multidrug resistance protein_ABCC	Xenobiotic transport / multidrug efflux	ACS47 (31 atoms)	−6.04 ± 0.29	17.26 ± 13.55	3.16×10^3 nM	−0.195 kcal/mol · atom^{-1}
P21441_PGPA-Multidrug resistance protein_ABCC	Xenobiotic transport / multidrug efflux	ACS48 (44 atoms)	−6.36 ± 0.36	7.53 ± 6.76	1.87×10^3 nM	−0.145 kcal/mol · atom^{-1}
P21441_PGPA-Multidrug resistance protein_ABCC	Xenobiotic transport / multidrug efflux	ACS51 (52 atoms)	−6.10 ± 0.22	4.65 ± 6.43	2.72×10^3 nM	−0.117 kcal/mol · atom^{-1}

P27116_Ornithine decarboxylase	Polyamine biosynthesis	ACS47 (31 atoms)	−6.54 ± 0.22	11.98 ± 11.98	1.63×10^3 nM	−0.211 kcal/mol · atom^{-1}
P27116_Ornithine decarboxylase	Polyamine biosynthesis	ACS48 (44 atoms)	−7.18 ± 0.29	6.32 ± 6.63	5.09×10^2 nM	−0.163 kcal/mol · atom^{-1}
P27116_Ornithine decarboxylase	Polyamine biosynthesis	ACS51 (52 atoms)	−6.94 ± 0.33	4.84 ± 2.17	7.49×10^2 nM	−0.133 kcal/mol · atom^{-1}
P36400_Cysteine proteinase B	Proteolysis / host–pathogen virulence	ACS47 (31 atoms)	−6.56 ± 0.33	5.39 ± 6.59	1.59×10^3 nM	−0.212 kcal/mol · atom^{-1}
P36400_Cysteine proteinase B	Proteolysis / host–pathogen virulence	ACS48 (44 atoms)	−6.98 ± 0.25	1.90 ± 1.08	7.15×10^2 nM	−0.159 kcal/mol · atom^{-1}
P36400_Cysteine proteinase B	Proteolysis / host–pathogen virulence	ACS51 (52 atoms)	−5.92 ± 0.23	1.64 ± 0.46	4.98×10^3 nM	−0.114 kcal/mol · atom^{-1}
P43152_HGPRT	Purine salvage pathway	ACS47 (31 atoms)	−6.40 ± 0.18	2.55 ± 1.21	2.10×10^3 nM	−0.207 kcal/mol · atom^{-1}
P43152_HGPRT	Purine salvage pathway	ACS48 (44 atoms)	−6.16 ± 0.22	1.97 ± 0.27	3.16×10^3 nM	−0.140 kcal/mol · atom^{-1}
P43152_HGPRT	Purine salvage pathway	ACS51 (52 atoms)	−7.08 ± 0.38	1.64 ± 0.58	6.39×10^2 nM	−0.136 kcal/mol · atom^{-1}
Q0GU43_Trypanothione reductase	Redox metabolism / trypanothione pathway	ACS47 (31 atoms)	−6.62 ± 0.54	3.39 ± 2.90	1.38×10^3 nM	−0.214 kcal/mol · atom^{-1}
Q0GU43_Trypanothione reductase	Redox metabolism / trypanothione pathway	ACS48 (44 atoms)	−6.60 ± 0.33	17.92 ± 10.00	1.47×10^3 nM	−0.150 kcal/mol · atom^{-1}
Q0GU43_Trypanothione reductase	Redox metabolism / trypanothione pathway	ACS51 (52 atoms)	−6.68 ± 0.32	16.45 ± 12.14	1.33×10^3 nM	−0.128 kcal/mol · atom^{-1}
Q0P0L8_Miltefosine transporter beta subunit_LdRos3	Phospholipid translocation / drug resistance	ACS47 (31 atoms)	−6.82 ± 0.26	1.47 ± 0.48	1.06×10^3 nM	−0.220 kcal/mol · atom^{-1}
Q0P0L8_Miltefosine transporter beta subunit_LdRos3	Phospholipid translocation / drug resistance	ACS48 (44 atoms)	−6.96 ± 0.20	1.94 ± 0.51	7.88×10^2 nM	−0.158 kcal/mol · atom^{-1}
Q0P0L8_Miltefosine transporter beta subunit_LdRos3	Phospholipid translocation / drug resistance	ACS51 (52 atoms)	−6.32 ± 0.15	6.49 ± 4.46	2.22×10^3 nM	−0.122 kcal/mol · atom^{-1}

Q4Q0M3_NDH2-Putative NADH dehydrogenase	Mitochondrial electron transport / NADH oxidation	ACS47 (31 atoms)	−6.88 ± 0.29	28.11 ± 6.15	1.27 × 10^3 nM	−0.222 kcal/mol · $atom^{-1}$
Q4Q0M3_NDH2-Putative NADH dehydrogenase	Mitochondrial electron transport / NADH oxidation	ACS48 (44 atoms)	−7.22 ± 0.09	26.29 ± 7.42	5.30 × 10^2 nM	−0.164 kcal/mol · $atom^{-1}$
Q4Q0M3_NDH2-Putative NADH dehydrogenase	Mitochondrial electron transport / NADH oxidation	ACS51 (52 atoms)	−7.00 ± 0.23	5.20 ± 6.83	8.29 × 10^2 nM	−0.135 kcal/mol · $atom^{-1}$
Q4Q3K2_Cytochrome c peroxidase mitochondrial_complex II	Mitochondrial oxidative stress / electron transport	ACS47 (31 atoms)	−8.56 ± 0.17	3.65 ± 1.77	5.04 × 10^1 nM	−0.276 kcal/mol · $atom^{-1}$
Q4Q3K2_Cytochrome c peroxidase mitochondrial_complex II	Mitochondrial oxidative stress / electron transport	ACS48 (44 atoms)	−8.82 ± 0.38	2.69 ± 0.39	2.06 × 10^1 nM	−0.200 kcal/mol · $atom^{-1}$
Q4Q3K2_Cytochrome c peroxidase mitochondrial_complex II	Mitochondrial oxidative stress / electron transport	ACS51 (52 atoms)	−8.44 ± 0.26	1.47 ± 0.49	6.58 × 10^1 nM	−0.162 kcal/mol · $atom^{-1}$
Q4Q6R2_Aquaglyceroporin-AQP1	Glycerol and water transport / membrane permeation	ACS47 (31 atoms)	−7.58 ± 0.53	1.48 ± 0.38	2.79 × 10^2 nM	−0.245 kcal/mol · $atom^{-1}$
Q4Q6R2_Aquaglyceroporin-AQP1	Glycerol and water transport / membrane permeation	ACS48 (44 atoms)	−8.70 ± 0.29	2.29 ± 0.25	4.46 × 10^1 nM	−0.198 kcal/mol · $atom^{-1}$
Q4Q6R2_Aquaglyceroporin-AQP1	Glycerol and water transport / membrane permeation	ACS51 (52 atoms)	−8.00 ± 0.20	1.63 ± 0.45	1.38 × 10^2 nM	−0.154 kcal/mol · $atom^{-1}$
Q4Q8E9_MRPA-ABC-thiol transporter	Xenobiotic efflux / thiol-based detoxification	ACS47 (31 atoms)	−6.52 ± 0.38	2.65 ± 1.00	1.64 × 10^3	−0.210 kcal/mol · $atom^{-1}$
Q4Q8E9_MRPA-ABC-thiol transporter	Xenobiotic efflux / thiol-based detoxification	ACS48 (44 atoms)	−5.99 ± 0.27	11.96 ± 16.5	4.79 × 10^3	−0.136 kcal/mol · $atom^{-1}$

Q4Q8E9_MRPA-ABC-thiol transporter	Xenobiotic efflux / thiol-based detoxification	ACS51 (52 atoms)	−6.30 ± 0.36	19.17 ± 13.9	2.39×10^3	−0.121 kcal/mol · atom^{-1}
Q4QEW7_Dihydroorotate dehydrogenase_DHODH	Pyrimidine biosynthesis	ACS47 (31 atoms)	−6.96 ± 0.24	6.94 ± 7.10	7.94×10^2 nM	−0.225 kcal/mol · atom^{-1}
Q4QEW7_Dihydroorotate dehydrogenase_DHODH	Pyrimidine biosynthesis	ACS48 (44 atoms)	−7.06 ± 0.29	2.68 ± 0.39	6.06×10^2 nM	−0.160 kcal/mol · atom^{-1}
Q4QEW7_Dihydroorotate dehydrogenase_DHODH	Pyrimidine biosynthesis	ACS51 (52 atoms)	−6.64 ± 0.33	10.91 ± 5.49	1.40×10^3 nM	−0.128 kcal/mol · atom^{-1}
Q4QIU7_hemG_Protoporphyrinogen IX dehydrogenase	Heme biosynthesis	ACS47 (31 atoms)	−5.34 ± 0.22	7.00 ± 7.70	1.23×10^5	−0.172 kcal/mol · atom^{-1}
Q4QIU7_hemG_Protoporphyrinogen IX dehydrogenase	Heme biosynthesis	ACS48 (44 atoms)	−5.98 ± 0.08	1.63 ± 0.95	4.17×10^4	−0.136 kcal/mol · atom^{-1}
Q4QIU7_hemG_Protoporphyrinogen IX dehydrogenase	Heme biosynthesis	ACS51 (52 atoms)	−5.82 ± 0.19	1.30 ± 0.83	5.47×10^4	−0.112 kcal/mol · atom^{-1}
Q8T4Q0_NMT	Myristoylation / lipid modification	ACS47 (31 atoms)	−6.70 ± 0.18	1.91 ± 1.26	1.20×10^3	−0.216 kcal/mol · atom^{-1}
Q8T4Q0_NMT	Myristoylation / lipid modification	ACS48 (44 atoms)	−7.22 ± 0.29	1.99 ± 1.05	514	−0.164 kcal/mol · atom^{-1}
Q8T4Q0_NMT	Myristoylation / lipid modification	ACS51 (52 atoms)	−6.82 ± 0.23	6.86 ± 4.12	957	−0.131 kcal/mol · atom^{-1}
Q9NBV4_Nucleoside transporter 2-NT2	Nucleoside salvage and transport	ACS47 (31 atoms)	−6.74 ± 0.26	1.82 ± 1.11	1.10×10^3	−0.218 kcal/mol · atom^{-1}
Q9NBV4_Nucleoside transporter 2-NT2	Nucleoside salvage and transport	ACS48 (44 atoms)	−7.72 ± 0.20	5.96 ± 4.73	209	−0.176 kcal/mol · atom^{-1}
Q9NBV4_Nucleoside transporter 2-NT2	Nucleoside salvage and transport	ACS51 (52 atoms)	−7.18 ± 0.33	2.79 ± 1.72	517	−0.138 kcal/mol · atom^{-1}

Q7IS90_Superoxide dismutase	Oxidative stress response / Reactive oxygen detoxification	ACS47 (31 atoms)	−6.08 ± 0.29	3.60 ± 3.40	3.53×10^3	−0.196 kcal/mol · atom^{-1}
Q7IS90_Superoxide dismutase	Oxidative stress response / Reactive oxygen detoxification	ACS48 (44 atoms)	−6.14 ± 0.09	1.75 ± 0.38	3.09×10^3	−0.140 kcal/mol · atom^{-1}
Q7IS90_Superoxide dismutase	Oxidative stress response / Reactive oxygen detoxification	ACS51 (52 atoms)	−6.16 ± 0.29	2.00 ± 0.15	2.97×10^3	−0.118 kcal/mol · atom^{-1}
Q01782_Pteridine reductase 1	Pteridine/folate salvage	ACS47 (31 atoms)	−6.70 ± 0.31	1.94 ± 1.39	1.24×10^4	−0.216 kcal/mol · atom^{-1}
Q01782_Pteridine reductase 1	Pteridine/folate salvage	ACS48 (44 atoms)	−6.88 ± 0.18	1.97 ± 1.12	9.15×10^3	−0.156 kcal/mol · atom^{-1}
Q01782_Pteridine reductase 1	Pteridine/folate salvage	ACS51 (52 atoms)	−7.38 ± 0.11	1.34 ± 0.78	3.94×10^3	−0.142 kcal/mol · atom^{-1}
Q06034_Multidrug resistance protein 1_ABCB1	Drug efflux / Xenobiotic resistance	ACS47 (31 atoms)	−6.74 ± 0.33	11.63 ± 21.7	1.11×10^3	−0.218 kcal/mol · atom^{-1}
Q06034_Multidrug resistance protein 1_ABCB1	Drug efflux / Xenobiotic resistance	ACS48 (44 atoms)	−7.12 ± 0.12	1.85 ± 1.06	6.66×10^2	−0.162 kcal/mol · atom^{-1}
Q06034_Multidrug resistance protein 1_ABCB1	Drug efflux / Xenobiotic resistance	ACS51 (52 atoms)	−6.56 ± 0.20	2.34 ± 0.33	1.67×10^3	−0.126 kcal/mol · atom^{-1}
Q25264_AdoMetDC	Polyamine biosynthesis	ACS47 (31 atoms)	−6.98 ± 0.16	2.64 ± 1.01	8.06×10^2	−0.225 kcal/mol · atom^{-1}
Q25264_AdoMetDC	Polyamine biosynthesis	ACS48 (44 atoms)	−7.76 ± 0.24	1.83 ± 0.56	2.37×10^2	−0.176 kcal/mol · atom^{-1}
Q25264_AdoMetDC	Polyamine biosynthesis	ACS51 (52 atoms)	−7.26 ± 0.37	2.12 ± 0.63	4.28×10^2	−0.140 kcal/mol · atom^{-1}
Q27673_Leishmanolysin	Host–pathogen interaction / proteolysis	ACS47 (31 atoms)	−5.72 ± 0.29	2.46 ± 1.25	6.0×10^4	−0.185 kcal/mol · atom^{-1}
Q27673_Leishmanolysin	Host–pathogen interaction / proteolysis	ACS48 (44 atoms)	−6.44 ± 0.35	7.99 ± 6.80	1.7×10^4	−0.146 kcal/mol · atom^{-1}
Q27673_Leishmanolysin	Host–pathogen interaction / proteolysis	ACS51 (52 atoms)	−5.88 ± 0.23	1.56 ± 0.49	4.1×10^4	−0.113 kcal/mol · atom^{-1}
